# HAS3-induced extracellular vesicles from melanoma cells stimulate IHH mediated c-Myc upregulation via the hedgehog signaling pathway in target cells

**DOI:** 10.1007/s00018-019-03399-5

**Published:** 2019-12-09

**Authors:** Uma Thanigai Arasu, Ashik Jawahar Deen, Sanna Pasonen-Seppänen, Sami Heikkinen, Maciej Lalowski, Riikka Kärnä, Kai Härkönen, Petri Mäkinen, Elisa Lázaro-Ibáñez, Pia R-M Siljander, Sanna Oikari, Anna-Liisa Levonen, Kirsi Rilla

**Affiliations:** 1 Institute of Biomedicine, University of Eastern Finland, Kuopio, Finland; 2 A.I. Virtanen Institute for Molecular Sciences, University of Eastern Finland, Kuopio, Finland; 3 Institute of Clinical Medicine, University of Eastern Finland, Kuopio, Finland; 4 Faculty of Medicine, Biochemistry and Developmental Biology, Meilahti Clinical Proteomics Core Facility, HiLIFE, University of Helsinki, Helsinki, Finland; 5 Division of Pharmaceutical Biosciences, Faculty of Pharmacy, Centre for Drug Research, University of Helsinki, Helsinki, Finland; 6 EV Group and EV Core, Molecular and Integrative Biosciences Research Programme, Faculty of Biological and Environmental Sciences, University of Helsinki, Helsinki, Finland

**Keywords:** Extracellular vesicles, IHH, Hedgehog signaling, c-Myc, Claspin, Proliferation, Melanoma, Cancer, Hyaluronan, Hyaluronan synthase

## Abstract

Intercellular communication is fundamental to the survival and maintenance of all multicellular systems, whereas dysregulation of communication pathways can drive cancer progression. Extracellular vesicles (EVs) are mediators of cell-to-cell communication that regulate a variety of cellular processes involved in tumor progression. Overexpression of a specific plasma membrane enzyme, hyaluronan synthase 3 (HAS3), is one of the factors that can induce EV shedding. HAS3, and particularly its product hyaluronan (HA), are carried by EVs and are known to be associated with the tumorigenic properties of cancer cells. To elucidate the specific effects of cancerous, HAS3-induced EVs on target cells, normal human keratinocytes and melanoma cells were treated with EVs derived from GFP-HAS3 expressing metastatic melanoma cells. We found that the HA receptor CD44 participated in the regulation of EV binding to target cells. Furthermore, GFP-HAS3-positive EVs induced HA secretion, proliferation and invasion of target cells. Our results suggest that HAS3-EVs contains increased quantities of IHH, which activates the target cell hedgehog signaling cascade and leads to the activation of c-Myc and regulation of claspin expression. This signaling of IHH in HAS3-EVs resulted in increased cell proliferation. Claspin immunostaining correlated with HA content in human cutaneous melanocytic lesions, supporting our in vitro findings and suggesting a reciprocal regulation between claspin expression and HA synthesis. This study shows for the first time that EVs originating from HAS3 overexpressing cells carry mitogenic signals that induce proliferation and epithelial-to-mesenchymal transition in target cells. The study also identifies a novel feedback regulation between the hedgehog signaling pathway and HA metabolism in melanoma, mediated by EVs carrying HA and IHH.

**Electronic supplementary material:**

The online version of this article (10.1007/s00018-019-03399-5) contains supplementary material, which is available to authorized users.

## Introduction

Cancer cells depend on bidirectional communication between other cells and the extracellular environment for sustained growth, invasion and metastasis. Extracellular vesicles (EVs) represent one of the numerous pathways for cellular communication [1]. EVs are membrane-enclosed particles that are shed from the cell surface, carry bioactive molecules [2] and contribute to, for example, the communication between tumor cells and the extracellular matrix [3]. During the past decade, extensive research has demonstrated a role for EVs as effective intercellular messengers in cancer [4–6]. EVs can stimulate malignant transformation of target cells either by paracrine signaling or by fusion and internalization with the recipient cells [5]. The importance of EVs in cancer progression was first observed in 1980 when vesicles from highly metastatic melanoma cells (B16 mouse-F10) enabled poorly metastatic cells (mouse B16-F1) to metastasize to the lungs [7].

EVs are secreted from cells through different mechanisms including (1) exocytosis of multivesicular bodies via fusion with the plasma membrane, (2) direct budding from the plasma membrane, and (3) via the tips of filopodia or other plasma membrane protrusions [8]. We have previously shown that overexpression of HAS3 triggers growth of filopodia [9], which enhances EV shedding [10]. Hyaluronan synthases (HAS1-3) are plasma membrane enzymes responsible for the synthesis of hyaluronan (HA), which is involved in numerous cellular functions, such as proliferation and adhesion [11, 12]. Expression of HAS2 [13] and HAS3 [14, 15] are associated with malignant transformation and aggressive tumor growth. However, the specific features of HAS3-induced EVs and their role in cancer progression have yet to be studied.

Enhanced proliferation, accompanied by altered activity and expression of cell cycle proteins, is one of the hallmarks of cancer cells [16]. The commitment to a new round of cell division (G1/S phase) is the point where cell cycle molecules are altered in cancer cells enabling uncontrolled proliferation [17]. c-Myc is one of the prominent proto-oncogenes. When deregulated it enables oncogenic potential by promoting uncontrolled cell cycle and proliferation by directly regulating the cyclin-dependent kinases (CDKs) [18, 19]. Selective dependence on interphase CDKs explains the increased rounds of division in tumor cells [20]. c-Myc’s involvement in proliferation is reflected by its action in regulating growth-promoting genes [21] and CDK4 [19, 22].

Claspin is a nuclear protein involved in DNA replication and cellular homeostasis [23]. Expression of claspin in normal cells is hardly detectable at the G0/G1 phase, while it spikes at the S/G2 phase and drops during mitosis [24]. Claspin has a dual role in DNA replication; it can either promote or inhibit the process, depending on the circumstances. For example, while claspin initiates DNA replication during the G1/S phase, it can also inhibit DNA replication and thereby cell proliferation during DNA damage and repair. Claspin protein level directly corresponds to an increased or decreased proliferation rate [24, 25]. Apart from being an upstream regulator of claspin, c-Myc itself can also be regulated by numerous signal transduction pathways like hedgehog, Wnt, Notch, IGF and NFкB [21]. Constitutive activation of these pathways in cancer facilitates over-expression of c-Myc, as the cell traverses through cell cycle progression [16, 21].

Here we focus on hedgehog signaling (HH), a conserved pathway that plays an important role in embryonic development and in the progression of numerous cancers [26–28]. Binding of HH ligands such as Indian hedgehog (IHH), desert hedgehog (DHH) and sonic hedgehog (SHH) to the smoothened, frizzled class receptor (Smo) stimulates HH signaling and a cascade of downstream intracellular events that regulates cell survival, proliferation and differentiation [29]. It has been shown that HH ligands can be carried via EVs [30, 31]. In this study, we treated normal keratinocytes and melanoma cells with EVs originating from metastatic melanoma cells overexpressing GFP-HAS3 and investigated their possible effects on the target cell properties. We observed that GFP-HAS3 EVs carried IHH, HAS3, EGF, EGFR and CD44. Moreover, IHH in HAS3-EVs activated the HH signaling pathway, which subsequently triggered c-Myc activation, cell proliferation and epithelial-to-mesenchymal transition. c-Myc-induced proliferation of target cells was mediated by the regulation of claspin. This study shows for the first time that EVs originating from HAS3 overexpressing cells carry mitogenic signals that induce sustained proliferation and EMT in target cells.

## Results

### Characterization of EVs secreted by MV3 human metastatic melanoma cells

**Fig. 1:**
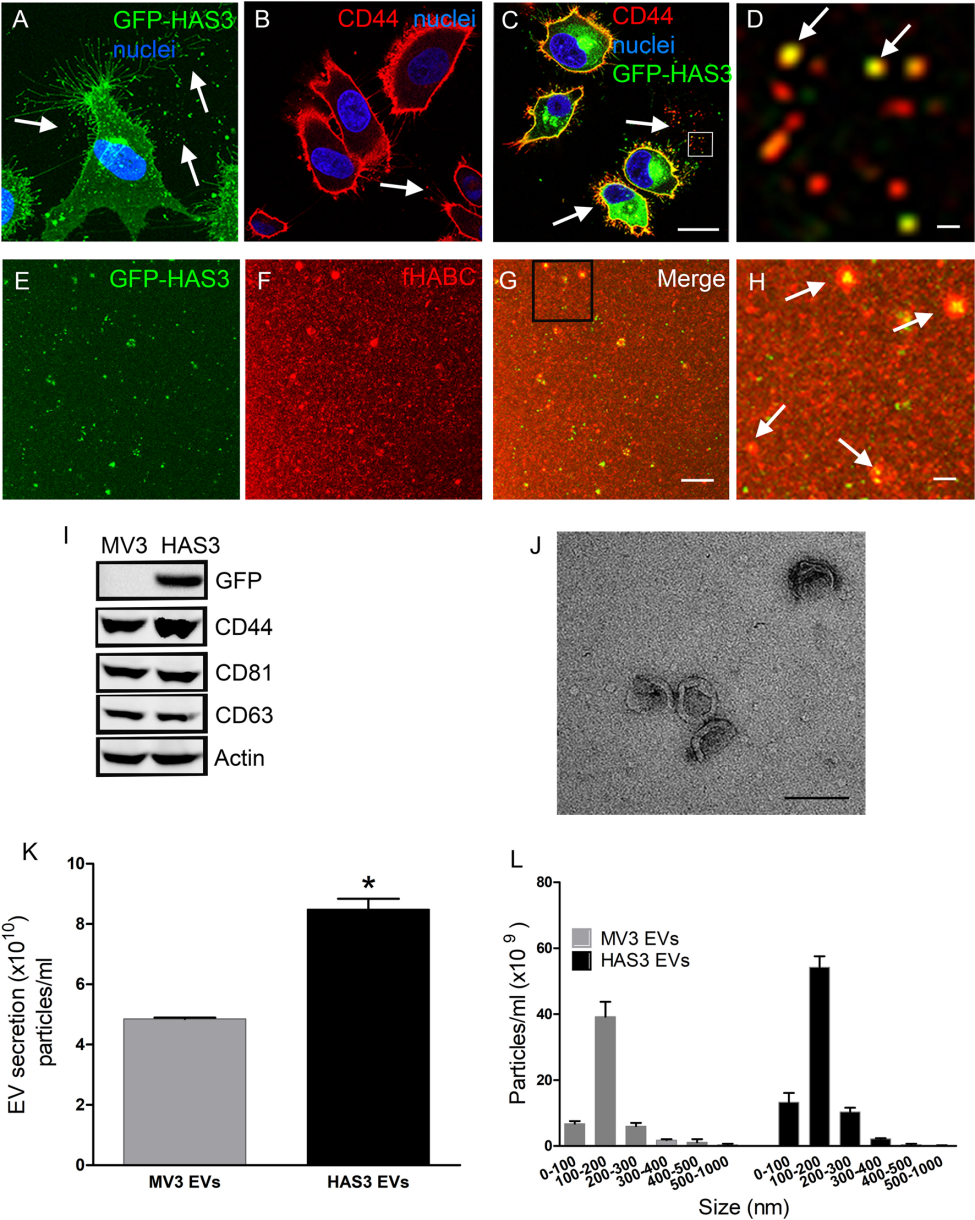
Characterization of EVs secreted by MV3 human metastatic melanoma cancer cells with stable, inducible expression of GFP-HAS3. **a** 3D projection of live GFP-HAS3 expressing cell grown in 3D collagen gel. Confocal sections of CD44-immunostained uninduced (**b**) and induced (**c**) MV3 cells and a higher magnification image of an area indicated by a white box in (**c**) is shown in (**d**). Panels **e**–**h** show HA staining of EV preparations isolated from GFP-HAS3 expressing cells, and **h** shows a higher magnification image of the area indicated by a black box in (**g**). **i** Western blotting of GFP-HAS3 detected with GFP antibody and other typical EV markers. Nanoparticle tracking analysis of EV levels (**j**) and size distribution (**k**) secreted from uninduced (MV3-EVs) and induced (HAS3-EVs) MV3 cells, and **l** transmission electron microscopy of EVs isolated from GFP-HAS3 expressing cells. Data represent mean ± S.E. from three independent experiments; *P < 0.05 (one-way ANOVA, Tukey’s test). Arrows in all panels indicate EVs. Scale bars represent 20 µm in (**c**), 2 µm in (**d**) and (**h**), 10 µm in (**g**) and 200 nm in (**j**)

EVs were isolated from MV3 human melanoma cells stably expressing GFP-HAS3, which has been characterized previously [32, 33]. To trap the GFP-HAS3-positive EVs for visualization in situ we cultured the MV3 cells in type I collagen matrix. Live GFP-HAS3 expressing cells are grown inside a 3D gel of type I collagen matrix showed a high number of GFP-HAS3-positive filopodia and EVs shedding into the surrounding matrix (Fig. 1a). EVs shed from GFP-HAS3 expressing MV3 cells were termed as HAS3-EVs. Immunostaining of the 3D cultures demonstrated that both doxycycline-induced and uninduced cells secreted CD44-positive EVs (arrows in Fig. 1b, c), and most of the HAS3-EVs displayed an association with CD44 immunostaining (Fig. 1d). The HA-positivity of HAS3-EVs was analyzed in isolated EV preparations with confocal microscopy (Fig. 1e–h). Staining with the HA probe, fluorescently labelled HA binding complex (fHABC), indicated that EVs of different sizes were coated with HA (arrows in Fig. 1h). HAS3-EVs carried GFP-HAS3, while uninduced cells with native-EVs (hereafter termed as MV3-EVs) were negative, as indicated with GFP immunostaining (Fig. 1i). EVs from both the induced and uninduced cells carried the HA receptor, CD44 and EV markers, such as CD81 and CD63, as well as actin (Fig. 1i). To analyze the EV secretion from these cells, we utilized NTA analysis, which showed a 75% increase in the total particle counts in the HAS3-EVs group, compared to MV3-EVs (Fig. 1k). No difference was detected in the size distribution between MV3- and HAS3-EVs, and their average diameter was 150 nm (Fig. 1l). TEM analysis of isolated preparations confirmed a typical structure of EVs (Fig. 1j). The EV secretion and size distribution were tested in one more melanoma cell line, C8161 with doxycycline induced GFP-HAS3. EVs from uninduced cells will be addressed as C8161-EVs while the EVs from induced cells will be addressed as C8161 HAS3-EVs. The NTA analysis revealed a 58% increase in the total particle counts in C8161-HAS3 EVs group compared to C8161-EVs (Suppl. Figure 2a) while the size distribution between the two groups remained unchanged (Suppl. Figure 2b).

### Binding of HAS3-EVs to target cells and their effect on HA secretion, proliferation and invasion

**Fig. 2:**
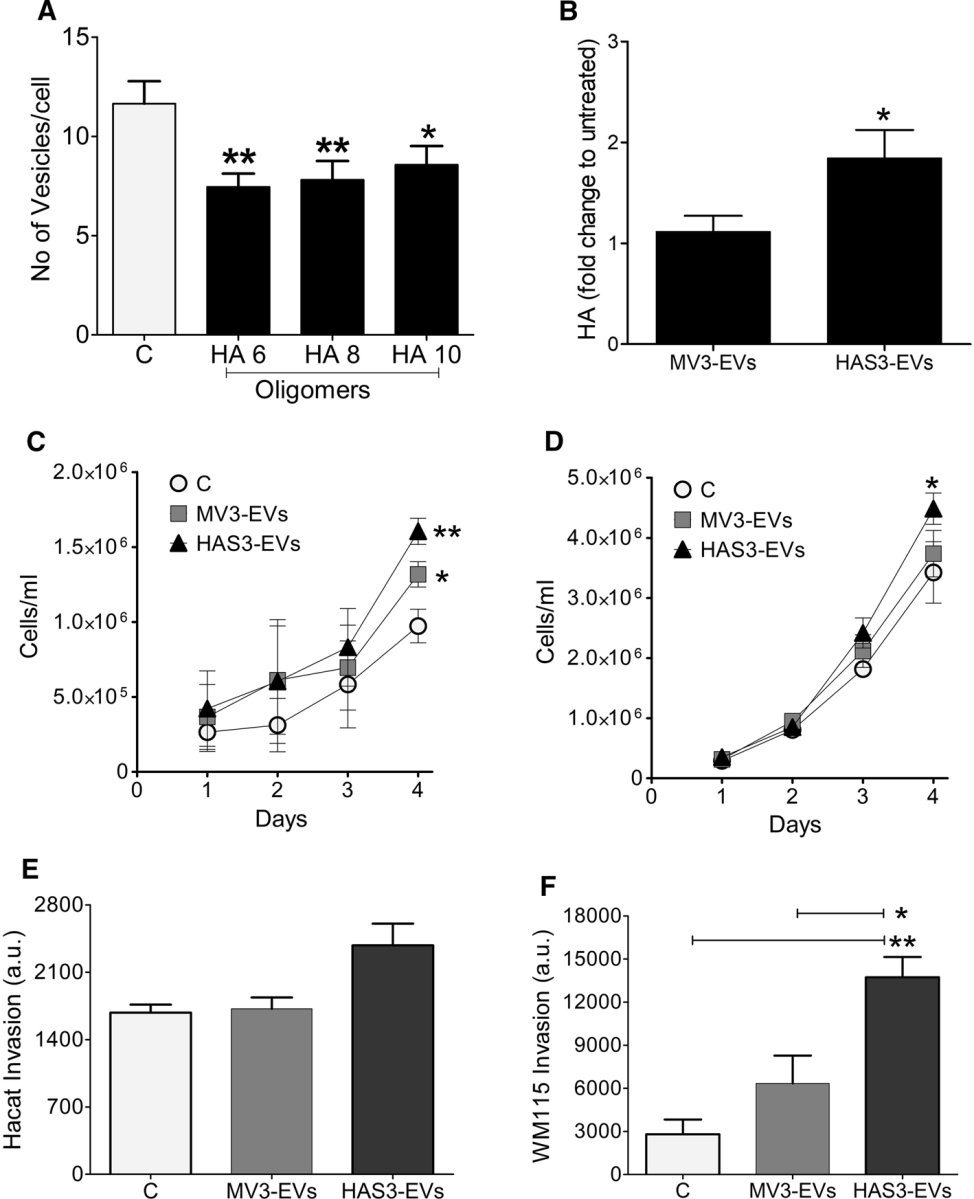
Binding of HAS3-EVs and their effect on target cell HA secretion, proliferation and invasion. **a** Effect of short HA oligosaccharides (6–10 monosaccharide units) on binding of EVs originating from HAS3-induced MV3 cells to HaCaT cells. **b** Effect of melanoma cell-derived EV treatment on HA secretion levels of HaCaT cells. HA secretion was quantified and normalized to RNA content of the cells. Effect of melanoma-derived EVs on the proliferation rate (**c**, **d**) and invasion (**e**, **f**) in HaCaT (**c**, **e**) and WM115 (**d**, **f**) cells, respectively. The data represent mean ± S.E. of four independent experiments. *P value < 0.05, **P value < 0.01, one-way ANOVA (Tukey’s test)

When a normal keratinocyte cell line, HaCaT, was treated with both EVs and HA oligomers of different lengths (HA6, HA8, HA10) (Suppl. Figure 1j, k), binding of EVs to the cell surface significantly decreased (Fig. 2a). HA oligomers are known to inhibit HA and CD44 interaction [34] and this showed that CD44 is one of the potential binding partners aiding in EV interaction with the cells. Treating HaCaT cells with HAS3-EVs increased HA secretion levels of the cells when compared to cells that were treated with MV3-EVs (Fig. 2b). Since increased cell proliferation is one of the earlier traits in cancer progression, we analyzed the effect of MV3- and HAS3-EVs on the proliferation rate of HaCaT and WM115, a primary melanoma cell line. Over a period of 4 days, both HaCaT (Fig. 2c) and WM115 (Fig. 2d) proliferation was significantly increased with MV3-EVs treatment, with a relatively higher rate in HAS3-EVs treatment. The proliferation rate of HaCaT cells when treated with EVs from a different melanoma cell line, C8161-GFP-HAS3 was observed to follow the same pattern. HaCaT cells treated with C8161 HAS3-EVs had significantly higher proliferation rate on day 4, compared to cells treated with C8161-EVs (Suppl. Figure 2c). We also assessed if the same trend was observed in cells invasion, after EV treatment using type I collagen as the matrix. While there was no significant change in the invasion of HaCaT cells (Fig. 2e), both MV3- and HAS3-EVs treatment-induced invasion in WM115 cells, with HAS3-EVs giving a statistically significant increase (Fig. 2f). The results showed that HAS3-EVs can promote oncogenic properties in the target cells, such as proliferation and invasion. Next, we investigated which factors could influence these changes.

### Downstream effects and key players of HAS3-EVs in target cells

**Fig. 3:**
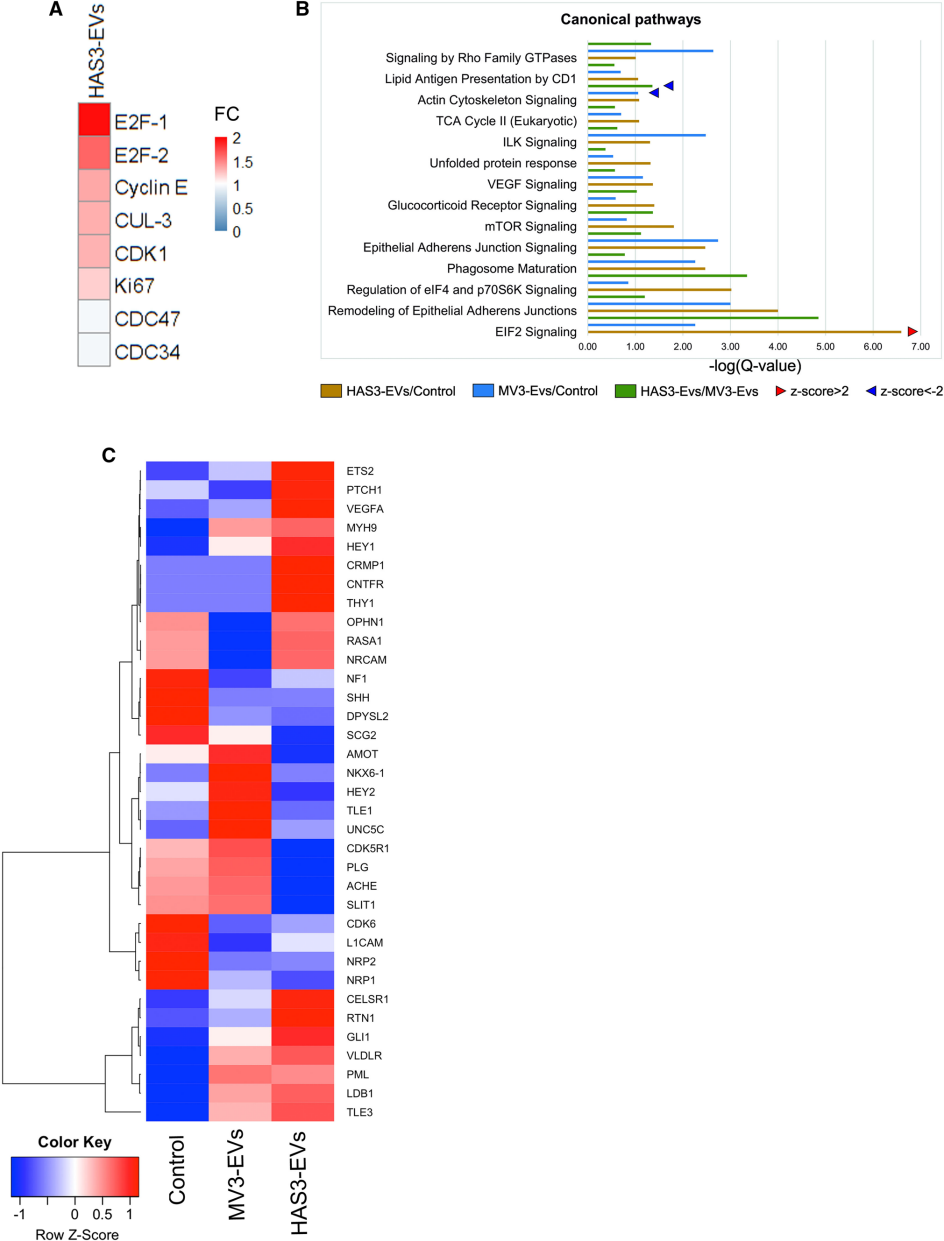
Downstream effects and keys players of HAS3-EVs in target cells. **a** Antibody array analysis showing the cell cycle protein expression of HaCaT cells, treated with HAS3-EVs compared to untreated control. The protein expression values of HaCaT cells treated with HAS3-EVs were charted based on fold change compared to untreated control. Six replicates of each protein on the array (*FC* fold change), **b** Differentially enriched canonical pathways of HaCaT cells proteome shared amid groups treated with MV3-EVs and HAS3-EVs, as compared to control. Arrow heads point to pathway activation (*z*-score > 2) or inhibition (*z*-score < -2), as predicted by IPA algorithms. **c** A heat map of row-wise scaled group mean mRNA expression of control HaCaT cells or cells treated with MV3- or HAS3-EVs (*N* = 3 per group) for genes involved in hedgehog signaling

Due to the increase in cell proliferation after MV3- and HAS3-EVs treatment, we next analyzed changes in cell cycle proteins using an antibody array to target 60 key proteins relevant for cell proliferation. Interestingly, HAS3-EVs treatment induced an increased level of proteins such as cyclin E, E2F1, E2F2, CDK1, Ki97 and cullin-3, which are involved in DNA transcription and cell division (Fig. 3a). These results gave the intriguing indication that EVs treatment causes changes in the cell proliferation rate, which prompted us to employ complementary high-throughput RNA sequencing and label-free quantitative proteomic techniques to identify key role players. From whole cell lysates of HaCaT treated with or without MV3- and HAS3-EVs, differentially expressed proteins were analyzed using quantitative proteomics. Pathway analysis was performed using IPA software, comparing MV3- and HAS3-EVs treatments with the untreated control (Fig. 3b; Suppl. Table 1). The top-ranked pathways included rearranged cytoskeleton, increased cell proliferation and remodeled epithelial adherens junctions, all of which were positively associated with the HAS3-EVs group, and to a lesser extent with MV3-EVs (Fig. 3b). This expression pattern shows that EVs have an impact on various processes in the cells promoting them towards uncontrolled proliferation and possibly EMT. Overall, from both protein array and mass spectrometry analyses, we could discern that E2F signaling and levels of other regulatory proteins involved in cell cycle progression were increased upon HAS3-EVs treatment.

Next, we wanted to identify the key driving factors through which HAS3-EVs affect these cellular processes. Using Western blotting, we compared the levels of c-Myc, NFκB-p65, CD44, EGFR and MAPK, which are involved in HA signaling and cell proliferation [35–39], in HaCaT cells treated with or without MV3- and HAS3-EVs. Furthermore, the levels of EMT markers such as E-cadherin and Slug were also analyzed (Suppl. Figure 1c). Although we could observe changes in many of the oncogenic and signaling proteins promoting cell proliferation, an increase in c-Myc expression in the HAS3-EVs group was profound and statistically significant. Additionally, the whole-cell proteome analysis of HaCaT cells showed claspin (Swiss-Prot ID: Q9HAW4, Suppl. Table 1) as one of the top hits in HAS3-EVs treatment. Claspin expression was significantly increased with HAS3-EVs (1.95-fold change) but not with MV3-EVs (0.86-fold change) (Suppl. Table 1). Thus, claspin could be one of the differentially expressed proteins between MV3- and HAS3-EVs treatment and we were interested to understand its role in HAS3-EVs induced cell proliferation. Involvement of claspin in cell cycle progression was also highlighted in the network analysis of the extracted proteome (CLSPN in Suppl. Figure 3). Interestingly, our RNA sequencing analysis showed that HaCaT cells treated with HAS3-EVs exhibited an enrichment of hedgehog signaling (HH) pathway genes such as *GLI1* and *PTCH1* when compared to untreated control and MV3-EVs treatment (Fig. 3c). Based on the above-described results, significantly elevated protein expression of claspin and mRNA levels of *GLI1* and *PTCH1* pinpoints that hedgehog signaling and claspin could be involved in HAS3-EVs mediated increased cell proliferation. Though not directly shown in our mass spectrometry or RNA-sequencing analyses, differential expression of c-Myc was observed with HAS3-EVs treatment with Western blotting. We could not find commonly affected key players in mRNA and protein expression in the treated HaCaT cells, thus differentially expressed targets that were related to cell proliferation were sorted out to characterize them further in our in vitro experiments. Therefore, we analyzed the possible connection between hedgehog signaling with c-Myc and claspin in HAS3-EVs mediated increase in cell proliferation.

### HAS3-EVs act through c-Myc and claspin in target cells to regulate proliferation

**Fig. 4:**
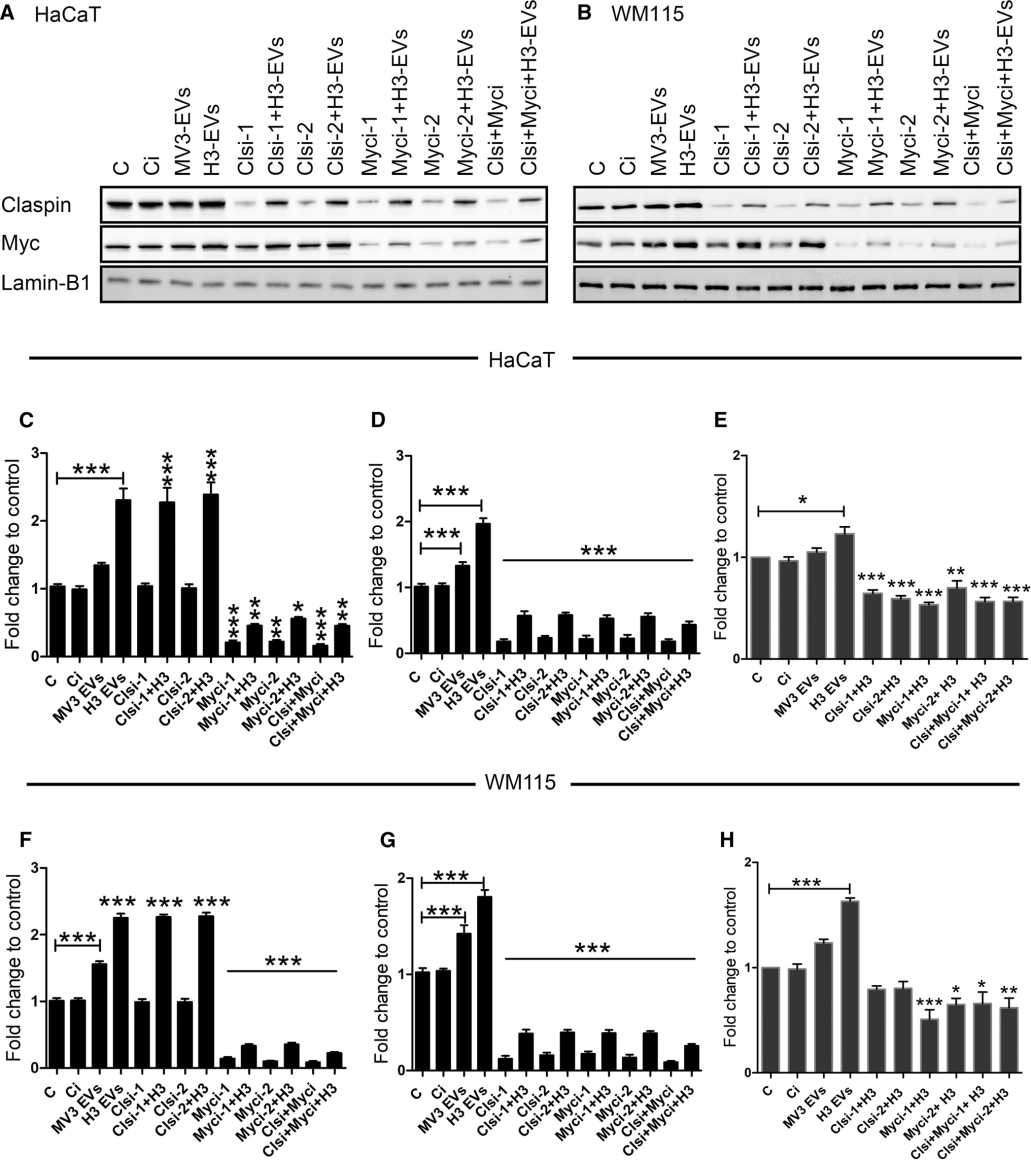
HAS3-EVs act through c-Myc and claspin in target cells to regulate proliferation. Western blots showing the expression of c-Myc and claspin in EVs- and siRNA-treated cells in HaCaT (**a**, **c**, **d**) and WM115 (**b**, **f**, **g**). Cell proliferation rate of HaCaT (**e**) and WM115 (**h**) cells with EVs- and siRNA-treatments. The statistical significance tests were done in comparison to control. The data represent mean ± S.E. of four independent experiments. *P value < 0.05, **P value < 0.01, ***P value < 0.001, one-way ANOVA (Tukey’s test). *C* control, *Ci* control siRNA (scrambled siRNA), *H3 or H3-EVs* HAS3-EVs, *Clsi and Myci* siRNAs for claspin and c-Myc

It has been previously reported that c-Myc and claspin regulate cell proliferation [24, 40] but their association with HH signaling and melanoma progression is largely unknown. Therefore, we studied the role of c-Myc and claspin on HaCaT and WM115 cell proliferation by siRNA-mediated knockdown. When treated with HAS3-EVs, c-Myc and claspin protein levels were significantly elevated, in both HaCaT (Fig. 4a, c, d) and WM115 cells (Fig. 4b, f, g). MV3-EVs treatment in both the cell types only led to minor changes in the expression of these proteins. Knocking down c-Myc and claspin with siRNAs significantly decreased their expression, while adding HAS3-EVs partially rescued it (Fig. 4a–d, f–g). Although knocking down c-Myc decreased the levels of claspin, the reciprocal effect was not observed. These results indicate that claspin expression was dependent on c-Myc activation for its expression while c-Myc protein expression was claspin-independent. Furthermore, HAS3-EVs had a direct role in c-Myc and claspin activation. Since c-Myc [21] and claspin [24] play a role in cell proliferation, we next studied the change in cell number 48 h post siRNA transfection. HAS3-EVs treated cells exhibited a higher proliferation rate compared to control cells (Fig. 4e, h). Also, HAS3-EVs treated cells with claspin and c-Myc knockdown showed a significantly lower proliferation rate (Fig. 4e, h). These results demonstrate that the increase in proliferation mediated by HAS3-EVs acted through c-Myc and claspin. Next, we aimed to resolve the specific activation mechanism of c-Myc by HAS3-EVs.

### HAS3-EVs induce c-Myc and EMT via HH signaling

**Fig. 5:**
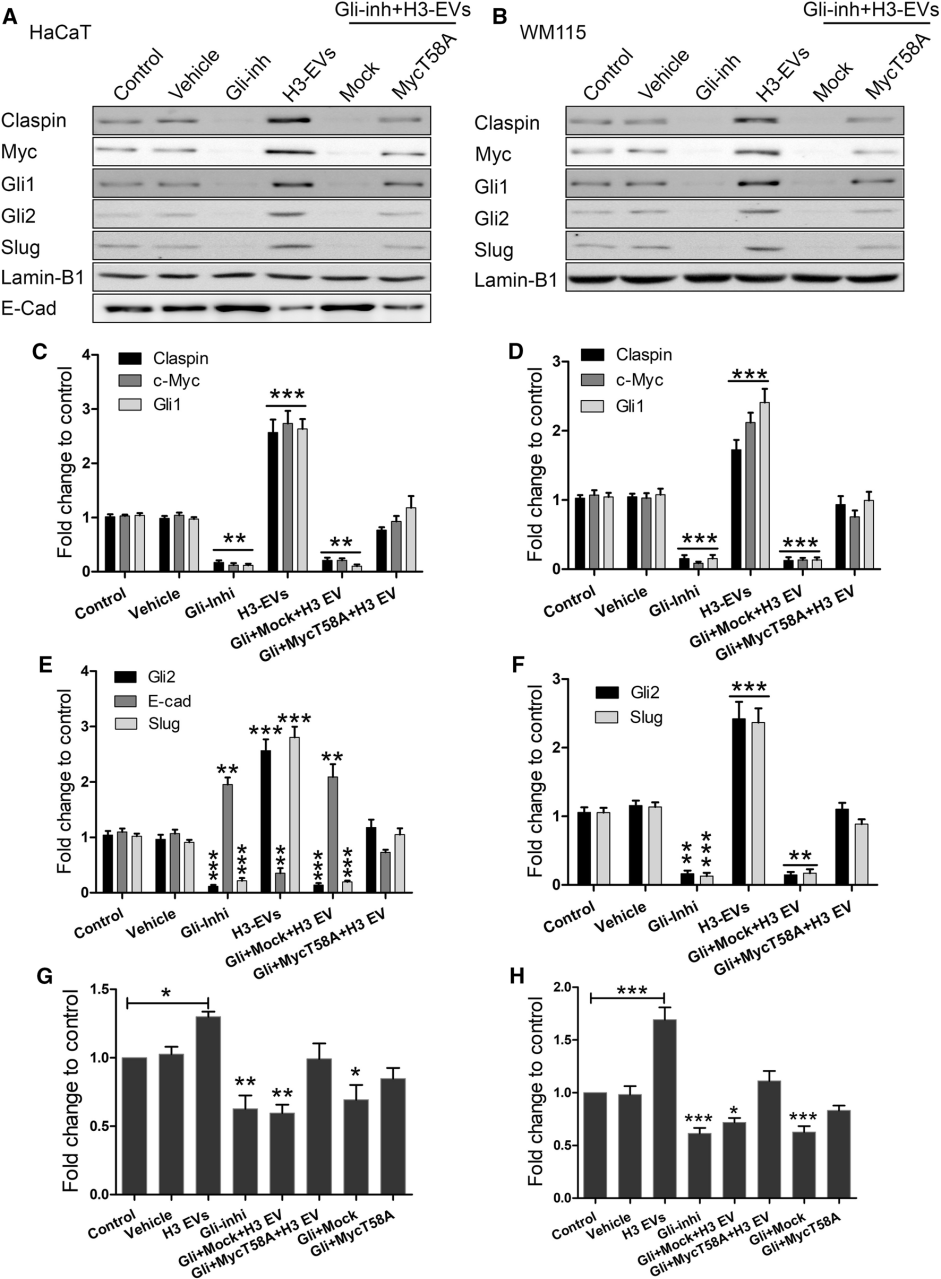
HAS3-EVs induce c-Myc and EMT by HH signaling. Protein expression of HH and EMT markers were tested in HaCaT (**a**, **c**, **e**) and WM115 cells (**b**, **d**, **f**). Both HaCaT and WM115 cells were treated with a Gli inhibitor, GANT58 and a constitutively expressing c-Myc T58A plasmid, along with HAS3-EVs. The impact of the treatments on proliferation was also tested in both HaCaT (**g**) and WM115 (**h**) cells. All the statistical significance tests were done in comparison to control. The data represent mean ± S.E. of four independent experiments. *P value < 0.05, **P value < 0.01, ***P value < 0.001, one-way ANOVA (Tukey’s test). *H3 or H3-EVs* HAS3-EVs, *Clsi and Myci* siRNAs for claspin and c-Myc, *E-cad* E-cadherin, *Gli-inhi or Gli* Gli inhibitor

Since HH signaling was enhanced by HAS3-EVs treatment (Fig. 3c), we investigated the direct effect of HH pathway members on c-Myc activation. To this end, the central HH pathway transcription factors, Gli-1 and -2 were targeted using a specific inhibitor called GANT58 [41]. Furthermore, the protein levels of Gli-1 and -2, along with claspin, c-Myc and EMT regulators such as slug and E-cadherin, were analyzed with Western blotting. Constitutively overexpressing c-Myc with a point mutation T58A activates its oncogenic functions and diminishes its ability to accelerate apoptosis [42, 43]. T58A mutation has also increased c-Myc protein stability to twofold [44]. We wanted to see whether Gli inhibition has any effect on c-Myc regulation of claspin and increased cell proliferation. To test it, we used constitutively activated c-Myc T58A, thus checking whether the mutant could reverse the effects of Gli inhibition. HaCaT (Fig. 5a, c, e) and WM115 cells (Fig. 5b, d, f) treated with HAS3-EVs showed an increase in Gli-1 and -2, c-Myc and claspin protein levels. Gli inhibitor effectively reversed the effect of HAS3-EVs by decreasing the expression of Gli-1 and -2, c-Myc and claspin. Moreover, c-Myc T58A but not mock transfection partially rescued the effect of HAS3-EVs on these protein levels (Fig. 5a–f). We also measured the proliferation rate as an end result of these treatments; the Gli inhibitor with or without HAS3-EVs decreased the proliferation, while c-Myc T58A treatment along with HAS3-EVs increased proliferation similar to control cells (Fig. 5g, h). These results suggest that HH signaling induced by HAS3-EVs activates c-Myc and claspin to regulate cell proliferation. In addition to enhanced proliferation rate, EMT is also a hallmark for cancer. We, therefore, studied an epithelial marker (E-cadherin) and mesenchymal marker (slug), to map out the extent of HAS3-EV treatment. As it can be seen from the results, HAS3-EVs treatment decreased E-cadherin (Fig. 5a, d) and increased slug levels (Fig. 5 a, d, b, f) (Suppl. Figure 1b–i). WM115 did not express E-cadherin endogenously. The results thus illustrate that HH signaling from HAS3-EVs is responsible for the increased proliferation rate and EMT via activation of c-Myc.

### IHH is the specific factor for HAS3-EV mediated c-Myc activation and claspin correlates with HA in human cutaneous melanocytic lesions

**Fig. 6:**
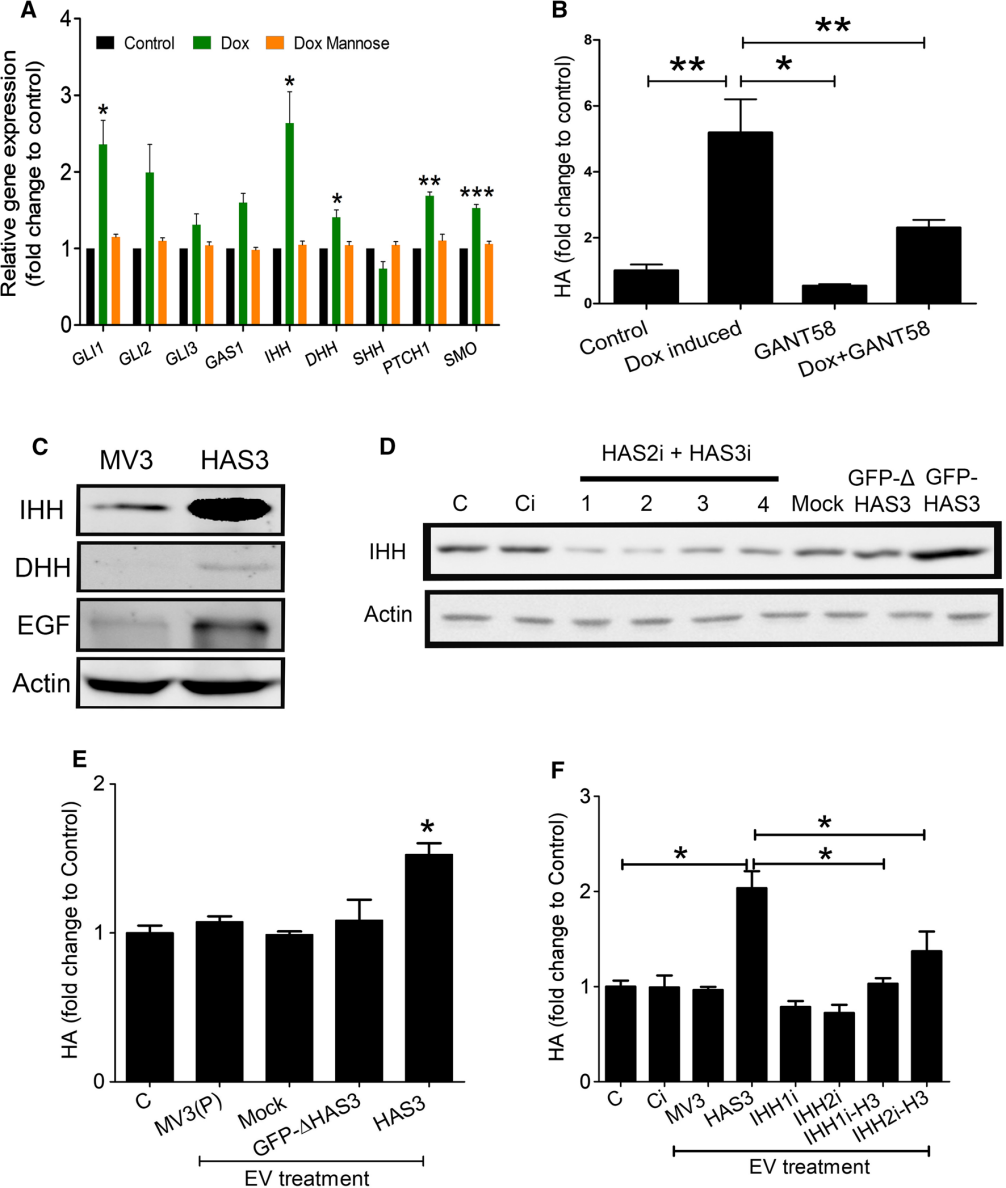
Presence of IHH in HAS3-EVs and feedback between HA and HH pathways. **a** q-PCR showing the RNA expression of HH genes in MV3-GFP-HAS3 cells. HA production was induced with doxycyclin (Dox) and inhibited with mannose. **b** HA levels were detected in MV3-GFP-HAS3 cells with or without doxycycline and GANT58. **c** Western blots showing expression of IHH, DHH and EGF mitogens in MV3- and HAS3-EV lysates. Actin was used as the loading control. **d** Western blots showing expression of IHH in EVs collected from MV3 parental cells treated with HAS2 + HAS3i cocktail, mock, GFP-ΔHAS3 [45] and GFP-HAS3 plasmid. **e** Effect of EVs collected from MV3 parental cells treated with mock, GFP-ΔHAS3 and GFP-HAS3 plasmid on HA secretion in HaCaT cells. **f** HA level in HaCaT cells after treatment with EVs collected from MV3-GFP-HAS3 cells treated with IHHi and dox induction (IHHi and IHHi-HAS3 EVs). HA secretion was normalized on RNA levels. Data represent mean ± S.E. of four independent experiments. *P value < 0.05, **P value < 0.01, one-way ANOVA (Tukey’s test). *C* control, *Ci* control siRNA (scrambled siRNA), *HAS3 or H3 or H3-EVs* HAS3-EVs, *MV3 (P) EVs* EVs from MV3 parental cells, *IHHi* IHH siRNAs, *HAS2i + HAS3i* HAS2 and HAS3 siRNAs, *Mock* pcDNA3 vector

Next, we wanted to identify which mitogen was responsible for the activation of the HH pathway and how it was associated with HAS3-EVs. There are three principal HH ligands—IHH, DHH and SHH, all of which act through Gli proteins [3]. Using doxycycline inducible GFP-HAS3 overexpressing MV3 cells, we analyzed the expression of HH genes by qPCR. Among the different genes that were tested, IHH, DHH, GLI1, PTCH1 and SMO expression were significantly increased in MV3 cells with GFP-HAS3 overexpression (Fig. 6a). This observation gave additional support to our RNA sequencing results, where we identified *GLI1* and *PTCH1* gene expression to be elevated with HAS3-EVs treatment in HaCaT cells (Fig. 3d). Additionally, the GFP-HAS3 overexpressing MV3 cells were also treated with mannose which reduces UDP-GlcNAc (UDP-*N*-acetyl glucosamine), a substrate used by HAS1-3 to synthesize HA [10]. Interestingly, mannose addition with GFP-HAS3 overexpression effectively reversed the increased expression of *IHH*, *DHH*, *GLI1*, *PTCH1* and *SMO* genes (Fig. 6a). Thus, it was clear that expression of HH signaling genes was associated with HA synthesis. To examine the putative relationship between HA synthesis and HH signaling, we again utilized MV3 cells with GFP-HAS3 overexpression by doxycycline induction. Of note, the addition of Gli inhibitor reduced HA secretion in the media when GFP-HAS3 expression was induced with doxycycline. Gli inhibitor did not influence HA secretion in the absence of doxycycline induction (Fig. 6b). We then analyzed the protein expression of IHH and DHH in MV3- and HAS3-EVs, alongside another well-known mitogen, epithelial growth factor (EGF). The results showed that HAS3-EVs contain a significantly higher IHH protein level, along with EGF, when compared with MV3-EVs. In fact, IHH level in HAS3-EVs was even more remarkable than EGF. However, DHH protein level was not different between the two groups (Fig. 6c). Therefore, we identified IHH to be one of the major mitogens present in HAS3-EVs.

**Fig. 7:**
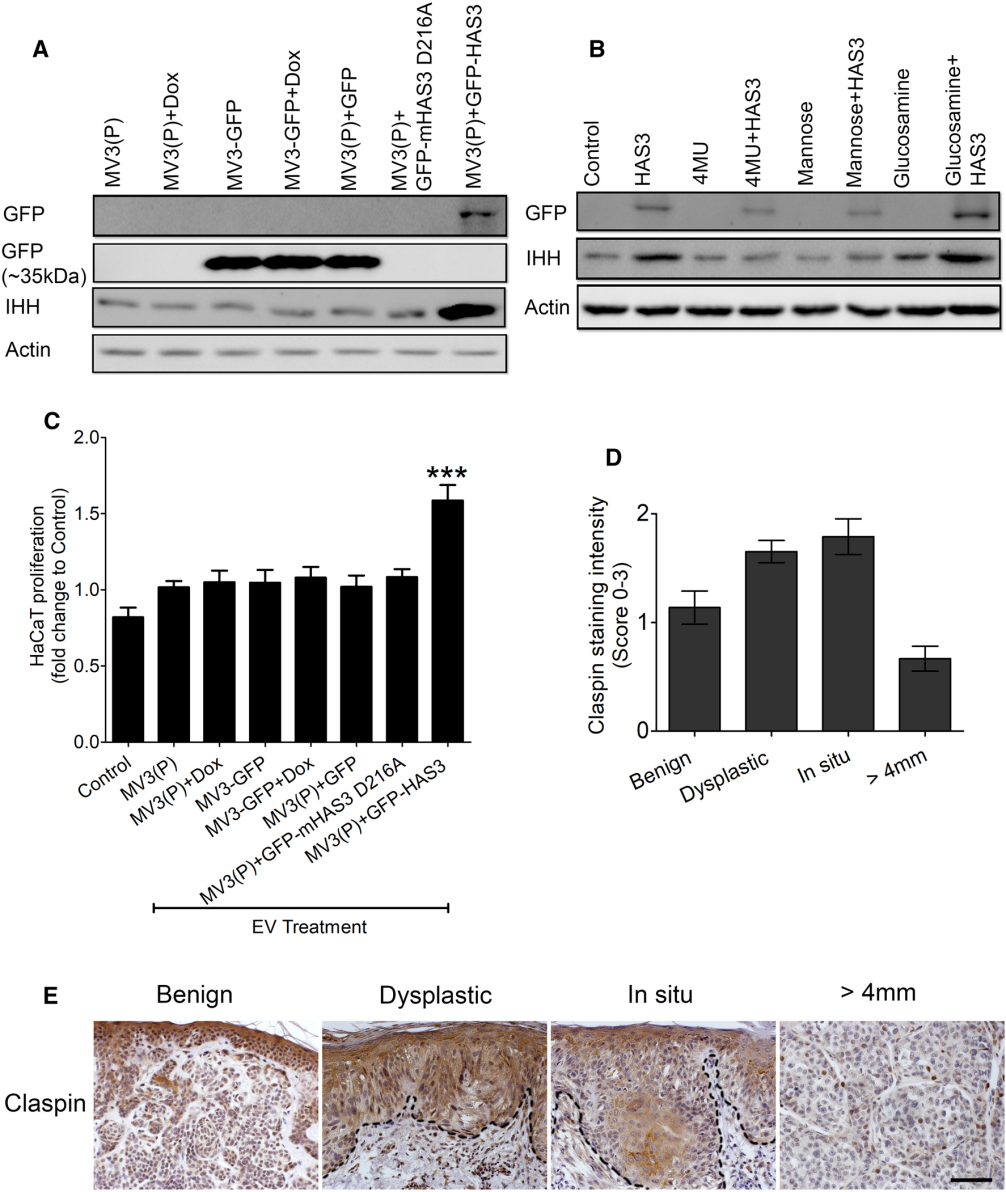
HA synthesis is associated with IHH secretion in HAS3-EVs. **a** Western blotting analysis of MV3 cells with GFP and GFP-mHAS3 D216A transfection. MV3 and MV3-GFP cells treated with or without doxycycline were also used as controls. The GFP band for HAS3 is near ~ 100 kDa while the band for only GFP is near ~ 35 kDa. **b** Western blotting analysis of MV3-HAS3 cells with or without 4MU, mannose and glucosamine treatments. Uninduced group was used as control. **c** 96 h HaCaT cell proliferation with or without treatment of EVs obtained from the described MV3 cell lines. Data represent mean ± S.E. of three independent experiments; **P value < 0.001, one-way ANOVA (Tukey’s test). In panels **a** and **c**, representative blots from 3 independent experiments are shown. **d**, **e** Melanoma tissue sections were stained to check for claspin immunoexpression, scale bar 100 µm. Data represent mean ± S.E. of 3–4 independent experiments in (**a**) and (**b**) and mean ± S.E. of 12–16 tissue sections score in (**d**). *P value < 0.05, **P value < 0.01, one-way ANOVA (Tukey’s test) in (**a**) and (**b**)

We next investigated whether HAS3 presence in plasma membrane and HA synthesis are associated with increased IHH secretion in EVs. MV3 parental cells were transiently transfected with an inactive GFP-ΔHAS3, which contains only the first 86 amino acids at N-terminus. Thus, the mutant protein cannot synthesize HA due to the absence of catalytic domain [45]. GFP-ΔHAS3 transfected cells showed a positive band near 35 kDa, whereas wildtype GFP-HAS3 was seen near 100 kDa in Western blotting (Suppl. Figure 3b). HA secretion was also not increased in GFP-ΔHAS3 transfected cells when compared to wildtype GFP-HAS3 group (Suppl. Figure 3c, d). Interestingly GFP-ΔHAS3 EVs did not show an increase in IHH protein level, unlike the wildtype GFP-HAS3 EVs (Fig. 6d). EVs collected from the GFP-ΔHAS3 transfected or parental MV3 cells did not increase HA secretion in HaCaT cells, when compared to cells treated with GFP-HAS3 EVs (Fig. 6e). Also, in MV3 parental cells, inhibition of endogenous HAS2 and HAS3 using a cocktail of siRNAs reduced IHH secretion in EVs (Fig. 6d). HAS2 and HAS3 inhibition also reduced HA secretion in MV3 cells (Suppl. Figure 4b). To further demonstrate the specificity of IHH as a stimulus in HAS3-EVs, siRNAs against IHH were used in MV3-GFP-HAS3 cells before isolating EVs. HaCaT cells were then treated with EVs from MV3-GFP-HAS3 cells with or without IHH siRNAs (or IHHi) and HA secretion was analyzed. The results showed a significant reduction in HA level in cells treated with IHHi-HAS3 EVs compared to cells treated with normal GFP-HAS3 EVs (Fig. 6f). This shows that IHH secretion in EVs is associated with HA synthesis and the released IHH in EVs influence HA synthesis in target cells. We have previously shown that HAS3 presence in plasma membrane and its release in EVs depends on its ability to synthesize HA. We have also demonstrated that HA synthesis by HAS3 is affected by D216A point mutation on its catalytic domain or by altering the availability of its substrates, UDP-GlcUA and UDP-GlcNAc using chemicals like 4MU, mannose and glucosamine [33, 46]. Though the point mutation was studied in mHAS3, it is important to pinpoint that there are 97% conserved amino acids shared between mHAS3 and human HAS3 protein sequences [47]. First, we transfected MV3 cells with GFP-mHAS3 D216A and treated MV3-HAS3 cells with 4MU, mannose and glucosamine. EVs were collected and examined for GFP and IHH immunostaining. As shown in (Fig. 7a), mHAS3 D216A was not present in EVs and did not increase IHH secretion, when compared to GFP-HAS3 transfection. While reducing UDP-GlcUA by 4MU and UDP-GlcNAc by mannose significantly blocked GFP-HAS3 presence in EVs, increasing UDP-GlcNAc by glucosamine enhanced GFP-HAS3 in EVs (Fig. 7b). IHH secretion was also increased in glucosamine treatment with GFP-HAS3 group (Fig. 7b). Glucosamine also had an effect without GFP-HAS3 overexpression, which could be partially explained by the fact that this chemical increases O-GlcNAc modification and activation of endogenous HAS3 and HAS2 [33, 48]. As the next step, we also analyzed changes in HaCaT cell proliferation with MV3-EVs containing GFP-mHAS3 D216A or GFP-HAS3. The results showed that only GFP-HAS3 EVs induced an increase in HaCaT proliferation (Fig. 7c), which is in line with our earlier observation (Fig. 2c). In all these experiments, doxycycline did not show any adverse effects (Suppl. Figure 2d–g). The above results show that HA synthesis is associated with the release of HAS3 and IHH in EVs. In addition, both HA synthesis and HH signaling have a positive feedback regulation on each other.

As proof of concept, we next wanted to know how claspin protein expression, as the downstream effector of HA-IHH-c-Myc signaling axis, correlates with HA content in different stages of cutaneous melanocytic lesions. Immunohistochemical staining indicated that keratinocytes and melanocytic cells were positive for intracellular claspin while it was almost negative in stroma (Fig. 7d). The staining intensity was moderate or weak in dysplastic melanocytic lesions and in situ melanomas, while the staining intensity in invasive melanomas was negative or weak (Fig. 7e). Interestingly, this staining pattern of claspin in different stages of melanoma corresponded to HA staining in melanoma progression as shown before [33, 49]. This suggests that during melanomagenesis claspin expression follows changes in HA content. Whether or not IHH and c-Myc follows a similar pattern of correlation to HA content during melanomagenesis remains to be studied in the future.

## Discussion

**Fig. 8:**
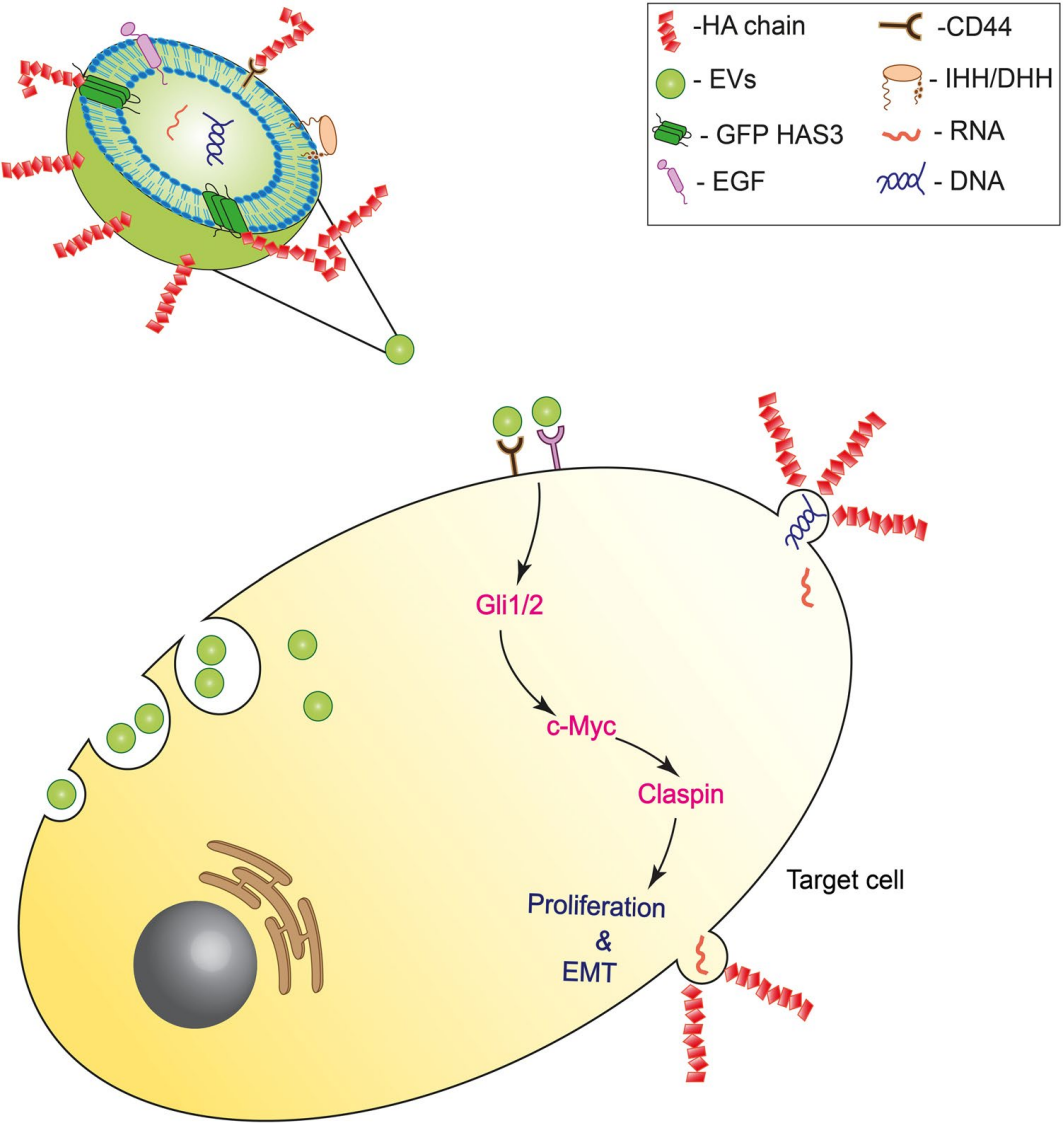
A schematic presentation of the mechanisms triggered by HAS3-induced EVs in target cells. HAS3-EVs from melanoma cells are possibly interacting with receptors such as CD44 present on the surface of recipient cells. As shown in the figure, direct fusion with the plasma membrane and endocytosis are also possible mechanisms of interaction. IHH present in the EVs activates the HH signaling cascade in the recipient cells, leading to the activation of c-Myc and claspin expression. This signaling cascade leads to an increase in proliferation and EMT in the recipient cells

Cutaneous melanoma is one of the deadliest cancers with nearly 80% mortality rate. It is a cancer with an aggressive clinical course which has recently been reported to have acquired resistance to existing therapies [50]. New targets for therapies and the development of new biomarkers depends on a better understanding of the signaling sequence and the biological events occurring in the tumor cells. Tumor cells secrete more EVs than normal cells and carry genetic and cellular information allowing communication with neighboring cells and the tumor microenvironment, either via paracrine or autocrine signaling [51]. EMT and pre–metastatic niche formation depend on tumor-derived EVs [2, 52, 53]. In this study, we demonstrated that EVs released from HAS3 overexpressing metastatic melanoma cells (MV3) interacted with keratinocyte (HaCaT) and melanoma (WM115) cells and induced proliferation and expression of EMT markers, similar to that in tumorigenesis (Fig. 8). HaCaT cells were used in this study since their low endogenous HA secretion facilitates analyses of transformative changes by EVs secreted from a melanoma cell line such as MV3 with high levels of HA. The MV3 cells released more EVs due to HAS3 overexpression and carried HAS3 and HA coat on the vesicles. Incubating HaCaT and WM115 cells with HAS3-EVs resulted in a higher proliferation rate than cells with no treatment, or cells treated with MV3-EVs. HAS3-EVs increased the expression of c-Myc, an oncogenic factor activated in many different cancers types [21, 54]. IHH present in HAS3-EVs acted as a stimulus upstream to c-Myc and we showed that the signaling axis extended to the activation of claspin. The IHH-c-Myc-claspin axis may help a normal keratinocyte (HaCaT) and a melanoma cell line (WM115) to gain tumorigenic potential, such as increased proliferation; this effect may be due to an enhanced G1/S phase transition and EMT. Since c-Myc and hedgehog signaling are independently known to increase tumorigenic progression of the cells [21, 27], the implicated cross-talk between the two different pathways, here acting as one, is of significance (Fig. 8). However, our data cannot exclude the possibility that c-Myc and IHH could have additional, unknown downstream effectors and this possibility remains to be investigated in the future. We have also shown that HA synthesis in melanoma cells is associated with the release of HAS3 and IHH in EVs and there is a positive feedback regulation between HA and HH pathways. Thus, we suggest that HA synthesis could be one of the stimulating factors for IHH secretion in melanoma cells. Future studies should focus on understanding the molecular mechanisms behind the feedback relationship shared by HA and HH pathways.

As a proto-oncogene, c-Myc plays an important role in the pathobiology of numerous cancers including melanoma. Being at the crossroads of numerous growth signaling pathways, c-Myc activates the transcription of several genes involved in cell growth, among which the E2F family of transcription factors have also been reported [55]. E2Fs are key factors in cell cycle progression with a direct control over cyclins and CDKs [56, 57]. Fernandez et al., reported that Myc interacts with E2F promoter and overexpression of Myc induces the expression of E2F genes [55, 58]. E2Fs are known to directly bind to and induce transcription of growth-promoting genes such as DHFR, DNA polymerase α, CDC6, ORC1, thymidine kinase, cyclin E, cyclin A and numerous other genes relevant for proliferation and differentiation [59–61]. Our antibody array results were complimentary to these findings; we found a twofold increase in E2F1 and > 1.5-fold increase in E2F2 in HaCaT cells treated with HAS3-EVs (Fig. 3a). Additionally, Cyclin E, CDK1 and the cell proliferation marker Ki67 were all increased upon HAS3-EVs treatment. c-Myc has been shown to regulate the expression of E2F transcription factors [55], which in turn regulate promoter activity of claspin and chk1 [62]. Our study showed that HAS3-EVs treated HaCaT and WM115 cells exhibited high levels of c-Myc and claspin and knocking them down decreased the proliferation rate. Furthermore, knocking down c-Myc decreased claspin expression while knocking down of claspin did not influence c-Myc levels. Bertolli et al., have shown that oncogenic stimulation causes replication stress, and oppositely, sustained E2F-dependent transcription allows the cells to decrease replicative stress and remove the block for cell proliferation [63]. During oncogenic transformation, cells could utilize claspin, because it helps to circumvent replicative stress [64]. However, in this study, we have shown that an increase in c-Myc and possibly E2F transcription factors are decisive factors in aiding enhanced cell proliferation due to IHH stimulus. Claspin plays a role downstream from c-Myc, perhaps regulating the replication stress due to increased proliferation. Nevertheless, the exact molecular mechanism behind claspin’s role remains to be explored.

Although we only investigated hedgehog signaling in this study, both hedgehog and MAPK pathways could be involved upstream of c-Myc, stimulating HAS3-EVs-mediated target cell proliferation. Crosstalk between MAPK and Gli was reported previously [65]. In this study, we observed that HAS3-EVs carried IHH and along with it, DHH and EGF as well. The high levels of IHH in HAS3-EVs were surprising, given that most of the reported studies on melanoma and other cancers have largely implicated the role of SHH in the process [66–68]. However, IHH is a well-known stimulus for self-renewal and enhanced proliferation in several cancers, including colorectal [69], lung [70], pancreatic [71], gastric [72], and T cell acute lymphoblastic leukemia [73]. Our results also point out that knocking down IHH in MV3-GFP-HAS3 cells negatively impacts the HAS3-EVs ability to enable HA secretion in HaCaT cells and downregulation of HAS2 and HAS3 in MV3 parental cells decreases IHH secretion in EVs. Thus, IHH could be an important mitogen in melanoma progression that has not been studied and it is interesting that HA signaling contributes to this phenomenon. The inhibition of Gli1/2 activity and the rescuing of c-Myc by transient transfection of MYC-T58A [74] demonstrated an involvement of the HH pathway in the HAS3-EVs treated cells. Activation of the HH pathway and increased c-Myc expression not only upregulated proliferation but also initiated EMT, which is important for cancer metastasis. The role of the HH pathway in EMT is well documented in cancer cells undergoing metastasis or tumor progression [75–79]. HH pathway signaling has been shown to have pathological effects in many cancer types [3]. Activation of the HH pathway increases cyclin D1 and B1 expression leading to an increase in proliferation in basal cell carcinoma [80].

Since MV3 is a metastatic melanoma cell line, it was of interest to analyze the relationship of HH gene expression and HA synthesis. Our results show that both the expression of HH genes and HAS3-mediated HA synthesis may form a positive feedback loop regulation. A similar positive feedback regulation between HA and other cancer promoting factors, such as ZEB1 [81] and Akt [82], has been identified, for example in breast cancer. Gli-1 and Gli-3 were shown to bind directly to the HAS2 promoter and regulate its transcription [83]. Although only HAS2 regulation is known so far, our results suggest that the HH pathway has a direct impact on HA synthesis, possibly by HAS3 regulation. We have previously shown that HA content increases in in situ melanoma but then declines in more advanced and invasive stages of melanoma, suggesting that HA may be more important in the early stages of cancer progression [33, 49]. In this work, as an end target of the HA-IHH-c-Myc-claspin signaling cascade, we have found that claspin follows a similar tissue staining pattern as HA during melanoma progression. However, it is unknown whether c-Myc and HH pathway factors Gli-1 and 2 also follow the same pattern of expression in different stages of melanoma.

About 40–50% of melanoma patients have mutations in valine at position 600 (Val600) of BRAF protein, which leads to aberrant activation of MAPK pathway [84]. Since MEK acts downstream to BRAF in the MAPK pathway, a combination of BRAF and MEK inhibitors such as vemurafenib, dabrafenib, trametinib and cobimetinib have improved the effect of treatment of patients with advanced and metastatic melanoma. Other classes of therapeutic drugs with improved efficacy that have gained attention in recent years are immunotherapeutic drugs such as checkpoint inhibitors and oncolytic viruses. Some of these drugs approved for advanced stages of melanoma include nivolumab and pembrolizumab (PD-1 inhibitors), ipilimumab (anti-CTLA4 antibody) and the oncolytic virus talimogene laherparepvec (TVEC). The increase in progression-free survival for these type of immunotherapy ranges from 2.9 to 11.5 months [85]. However, the challenge with these targeted therapeutics are the adverse effects and failure to improve patient survival to a significant extent [86, 87]. Also, almost 50% of patients do not respond to the immunotherapy option [85]. This demonstrates the urgent need to screen for novel biomarkers, to identify and target specific mutations and signaling pathways. EVs not only act as biomarkers in this regard but can also show how patients respond to a particular treatment. In peripheral blood, a gene signature of 15 differentially expressed genes, which includes MYC and CDK2, is predictive and prognostic to anti-CTLA4 immunotherapy treatment and 1 year overall survival in melanoma patients [88]. Tumor-derived EVs are abundant in plasma [89] and some of the differentially expressed genes described above could be applicable in melanoma EVs. Another study on melanoma patients pinpointed that lymphatic exudates contained enriched EVs with tumor-specific markers like tetraspanins, ICAM-1, Rab-GTPases and integrins. In fact, the tumor-specific markers are more enriched in lymph than in plasma, suggestive that lymph could be well suited for biomarker analysis in melanoma [90]. BRAF V600E oncogenic mutation is detected in lymph-derived melanoma EVs, and the increase in mutation frequency in patients correlates with risk of relapse [91]. These results indicate that EVs can be a powerful tool to assess melanoma progression and to predict therapy response. As shown in our study, one of the mitogens from hedgehog signaling pathway, IHH, is significantly enriched in melanoma derived HAS3-EVs. Considering the fact that HA content increases at the initial stages of melanoma [33, 49], and that there is a direct feedback regulation between HA synthesis and hedgehog signaling, one could speculate that melanoma EVs with enriched hedgehog mitogens like IHH may be a good predictor for the progression and therapy response at early stages of melanoma. In addition, it would be interesting to see if inhibitors of hedgehog signaling combined with the available immune therapeutics improve the survival of melanoma patients. Since HA coated EVs originate from HAS3 expressing cells [10], from primary cells such as mesothelial cells [92] and from human mesenchymal stem cells [93], their role as messengers in the extracellular space can be acknowledged. As shown in this study, HA-coated EVs are associated with the release of tumor promoting IHH mitogen from melanoma and these EVs could influence its surrounding microenvironment to melanoma progression. Future studies should focus on the mechanistic details on how HA synthesis mediates the release of IHH in EVs and how the secreted IHH promotes melanoma progression.

## Materials and methods

### Cell lines and culture conditions

MV3 parental cells, and the MV3 and C8161 cells stably overexpressing doxycycline-inducible GFP-HAS3 [32], the MV3 cells stably overexpressing GFP (MV3-GFP) and WM115 cells were cultured in DMEM medium with high glucose (4500 mg/l) (Euroclone, Milan, Italy). HaCaT cells were cultured in DMEM with low glucose (1000 mg/l) (Sigma-Aldrich, USA). All cells were supplemented with inactivated 10% fetal bovine serum (FBS) (HyClone, Thermo Scientific, Epsom, UK), 4 mM l-glutamine (Sigma-Aldrich, USA), 50 µg/ml streptomycin sulfate and 50 units/ml penicillin (Sigma-Aldrich, USA). The MV3 stable cell line was maintained in 50 µg/ml hygromycin and it was induced with 0.25 µg/ml doxycycline during experiments. To check the adverse effects of doxycycline on EV secretion and hyaluronan synthesis, MV3 and MV3-GFP cells were used (Suppl. Figure 2d–g).

EV-depleted FBS used for EV isolation experiments was prepared by ultracentrifugation at 110,000 × g for 16 h followed by sterile filtering with 0.22 µm filters (Minsart, Sartorius, Stedim, Biotech, Goettingen, Germany).

### EV isolation

MV3 and C8161 cells (3.2 × 10^6^) were plated in 15 cm cell culture dishes and incubated for 24 h to reach 50% confluency. The complete media was replaced with EV-free media (16 ml/dish) and collected after 48 h of incubation. To remove cell debris, the media was centrifuged at 1000 × g for 10 min at 4**°** C. Next, the supernatant was transferred into new tubes and centrifuged at 1200 × g for 20 min at 4**°** C. Finally, the supernatant was ultra-centrifuged at 110.000 × g for 90 min at 4**°** C and the pellet was dissolved in PBS (sterile filtered in 0.22 µM filter). The EV preparations were stored at − 80**°** C.

EVs isolated from MV3 cells stably overexpressing doxycycline-inducible GFP-HAS3 without dox induction are called as “MV3-EVs” and EVs collected after doxycycline induction are called as “HAS3-EVs”. EVs isolated from C8161 cells stably overexpressing doxycycline-inducible GFP-HAS3 without dox induction are called as “C8161-EVs” and EVs collected after doxycycline induction are called as “C8161-HAS3 EVs”. EVs isolated from parental melanoma MV3 cells are called as “MV3 parental EVs”. EVs isolated from cells after siRNA treatments for IHH are called as “IHHi EVs” and the same with dox induction are labelled as “IHHi-HAS3 EVs”. Similarly, EVs isolated from cells with the siRNA cocktail against HAS2 and HAS3 are called as “HAS2 + 3i EVs”. EVs isolated from MV3 cells with transient transfection of GFP-ΔHAS3 are called as “ΔHAS3 EVs”. The nomenclature is explained in the table (Table 1).

**Table 1:** Nomenclature of different types of EVs and cell lines used in this study

Cell line	Treatment	Collected EVs and their nomenclature
MV3-GFP-HAS3	No dox induction	MV3 EVs
MV3-GFP-HAS3	dox induction	HAS3 EVs
C8161-GFP-HAS3	No dox induction	C8161 EVs
C8161-GFP-HAS3	dox induction	C8161 HAS3 EVs
MV3 parental	No treatment	MV3 (P) EVs
MV3 parental	GFP-ΔHAS3 transfection	ΔHAS3 EVs
MV3 parental	HAS2 + 3i transfection	HAS2 + 3i EVs
MV3-GFP-HAS3	IHHi transfection	IHHi EVs
MV3-GFP-HAS3	IHHi transfection and dox induction	IHHi-HAS3 EVs

### Nanoparticle tracking analysis

The concentration and size distribution of EVs collected from MV3 cells were analyzed using a Nanoparticle Tracking analyzer (NTA) (Malvern Instruments Ltd., Malvern, UK) with an NS300 view unit. The settings used for data acquisition were as follows: camera level 13, acquisition time 30 s and detection threshold 3, kept constant during measurements. Analysis was done using the NTA 3.1 software (Nanosight, Amesbury, UK).

### Transfection

For transfections, cells were seeded in a 12 well plate (HaCaT: 11 × 10^4^ cells/well, WM115: 16 × 10^4^ cells/well, MV3: 1.1 × 10^4^), 6 cm dishes (HaCaT: 7 × 10^5^ cells/dish, WM115: 1.1 × 10^6^ cells/dish) and 15 cm dishes (MV3: 3 × 10^6^ cells/dish, MV3-GFP HAS3: 3.2  × 10^6^ cells/dish). Once they reached 40–50% confluency, they were transfected with plasmid DNA or siRNAs.

Plasmid transfection was done with Lipofectamine 3000 (Invitrogen) and siRNA transfections were done with Lipofectamine RNAiMAX (Invitrogen), according to the manufacturers’ instructions. c-Myc T58A (Addgene, USA), EGFP-C1 (Clontech, USA), pcDNA, GFP-mHAS3 D216A [46] and GFP-ΔHAS3 plasmids were used in 12 well, 6 cm and 15 cm plate formats. Predesigned siRNAs targeting claspin: siRNA-1 (60 nM) (ID: s34330), siRNA-2 (60 nM) (ID: s34332), c-Myc: siRNA-1 (HaCaT: 30 nM, WM115: 60 nM) (ID: s9130), siRNA-2 (HaCaT: 30 nM, WM115: 60 nM) (ID: s9131), IHH: siRNA-1(MV3-GFP HAS3: 50 nM) (ID: s7257), siRNA-2 (MV3-GFP HAS3: 50 nM) (ID: s7258), HAS2: siRNA-1 (MV3: 50 nM) (ID: s6457), siRNA-2 (MV3: 50 nM) (ID: s6459) and HAS3: siRNA-1 (MV3-GFP HAS3: 50 nM) (ID: s194495), siRNA-2 (MV3-GFP HAS3: 50 nM) (ID: s6460) were purchased from Ambion, Thermo Fisher Scientific Waltham, MA, USA. Scrambled (control) siRNA (30 nM) from Origene (ref no: SR30004) was used. The cell media containing the transfection mix was removed after 24 h and the cells were either treated with EVs for 24 h or re-plated for proliferation or treated for 48 h with EV-free serum media for collecting EVs.

### Treatments

Both MV3- and HAS3-EVs were diluted to a standard 200 µg/ml protein concentration in PBS. MV3- and HAS3-EVs were added to the target cells for treatment using a calculation of 120 µg protein to a seeding density of 0.3 × 10^6^ cells, for example in a 6-well growth area. For culture plates with higher growth area, the amount of EVs were calculated according to the corresponding seeding density i.e. 320 µg for 6 cm dish with 0.8 × 10^6^ seeded cells and 0.8 mg for 10 cm dish with 2.0 × 10^6^ seeded cells. The final concentration for Gli inhibitor, GANT58 (Sigma-Aldrich, USA) was 10 µM in HaCaT cells, 1 µM in WM115 cells and 5 µM in MV3 EGFP HAS3 cells. In some experiments, 1 mM Glucosamine (Sigma), 20 mM Mannose (Sigma,) and 0.5 mM 4MU (Sigma) were used. HaCaT cells were treated with hyaluronan oligosaccharides of various lengths (HA6, HA8 and HA10 (Seikagaku Kogyo Co., Tokyo, Japan) at a final concentration of 0.1, 0.2 and 0.5 mg/ml.

### Transmission electron microscopy (TEM)

The EV preparations (10 µl) isolated from MV3 cell culture media were layered onto carbon-coated glow-discharged nickel grids (Agar Scientific Ltd, Sanstedt, UK). Grids were fixed in 2% paraformaldehyde for 10 min, contrasted using 2% neutral uranyl acetate (UA) for 10–15 min in the dark and embedded in 1.8% methyl cellulose (25 Ctp)/0.4% UA. Samples were imaged using a JEOL JEM 2100F transmission electron microscope (Jeol Ltd, Tokyo, Japan) operated at 200 kV.

### Confocal microscopy

Fluorescent images were taken with 40 × (NA 1.3) and 63 × (NA 1.4) oil objectives on a Zeiss Axio Observer equipped with a Zeiss LSM 700 confocal module (Carl Zeiss Microimaging GmBH, Jena, Germany) and a Zeiss XL-LSM S1 incubator with temperature and CO_2_ control. ZEN software (Carl Zeiss Microimaging GmBH) was used for image processing and 3D rendering.

### Cell lysis and Western blotting

Cells (HaCaT and WM115) grown in 6 cm dishes were treated with EVs for 24 h and lysed using NE-PER™ kit (Thermo Scientific) to isolate nuclear and cytoplasmic fractions. Protein content was determined using a Pierce BCA protein assay kit (Thermo Scientific, Epsom, UK). The proteins in the cell fractions were separated using a 10% SDS-PAGE and transferred to nitrocellulose membranes (Protran, Whatman), blocked with 5% bovine serum albumin (BSA) in Tris buffered saline containing 0.1% Tween 20 (TBST) or 5% skimmed milk-TBST for 1 h at room temperature and then incubated overnight with anti-claspin (1:2000, 5% BSA-TBST) (Proteintech, USA), anti-c-Myc (1:500, 5% BSA-TBST) (Santa Cruz, USA), anti-CD63 (1:1000, 5% BSA-TBST) (Abcam, UK), anti-Actin (1:2000, 5% milk-TBST) (Santa Cruz), anti-lamin β1 (1:2000, 5% milk-TBST) (Abcam), anti-Gli1 (1:1000, 5% BSA-TBST) (Abcam), anti-Gli2 (1:1000, 5% BSA-TBST) (Proteintech), anti-DHH (1:1000, 5% BSA-TBST) (Abcam), anti-IHH (1:1000, 5% BSA-TBST) (Abcam), anti-MAPK p42/44 (1:1000, 2% BSA-TBST) (Cell Signaling), anti-NFκB p65 (1:200, 1% BSA-TBST) (Santa Cruz), anti-EGFR (1:1000, 5% BSA-TBST) (Cell Signaling), anti-CD44 (1:2000, 1% BSA-TBST) (Chemicon, Temecula, CA), anti-EGF (1:1000, 5% BSA-TBST) (Abcam), anti-GFP (1:2000, 5% BSA-TBST) (Invitrogen) and anti-Calnexin (1:2000, 5% BSA-TBST) (Cell Signaling). Alexa-Fluor plus 555 anti-mouse or Alexa-Fluor plus 680 anti-rabbit secondary antibodies in 2% milk-TBST (1:10,000 dilution) (Thermo Scientific) were used. The membranes were washed three times for 5 min with TBST after antibody incubations and visualized using a BioRad ChemiDoc scanner.

### RNA isolation and q-PCR

The cells were lysed using NucleoZOL (Macherey–Nagel, Germany) and RNA extraction was done according to the manufacturer’s instructions. For q-PCR, the primer sequences used are mentioned in supplementary Table 2. Roche FastStart Universal SYBR Master mix was used. Relative mRNA levels were calculated according to 2^(− ΔΔCT) method with GAPDH used as the housekeeping gene.

### Hyaluronan assay

MV3-GFP-HAS3 cells grown in six-well plates (2 × 10^5^ cells/well) were induced with doxycycline (0.25 µg/ml). 24 h post seeding, the cells were treated with mannose or GANT58 for 6 h. The growth medium was then collected and stored at − 20 **°**C. Culture medium was also collected from 1 × 10^5^ HaCaT cells grown in 12 well plate, treated with MV3- or HAS3-EVs for 24 h. The cells were then lysed using NucleoZOL (Macherey–Nagel, Germany) and RNA extraction was carried out. In some cases, cell numbers were also counted. The HA content in the culture medium was analyzed using a previously established enzyme-linked sorbent assay [94]. The HA content was normalized to RNA concentration.

### RNA sequencing and data analysis

HaCaT cells were grown in six well plates (4.5 × 10^5^ cells/well) and treated with MV3- and HAS3-EVs for 24 h. Cells were lysed, and RNA was extracted using a high pure RNA isolation kit (Roche). The isolated RNA samples (*N* = 3 per group) were then sent for RNA-seq library generation (Illumina TruSeq stranded mRNA library preparation kit) and single-end sequencing using Illumina Hi seq (Illumina HiSeq 3000 instrument) at the Finnish Functional Genomics Centre, Turku center for biotechnology (Finland).

The 50 nt RNA-seq reads were quality controlled using FastQC (version 0.10.1) and trimmed with Trimmomatic (version 0.33) [95] using long TruSeq 3-SE.v2 adapters with the essential settings: ILLUMINACLIP:0:30:10, LEADING:3, TRAILING:3, MINLEN:35. Trimmed reads originating from mitochondrial DNA or ribosomal RNA, or composed of a single nucleotide, were then removed using bowtie2 (version 2.2.3) [96]. The remaining reads were aligned to the Gencode human transcriptome version 24 basic (for genome version hg38) using Tophap2 (version 2.0.13) [97] with essential non-default settings: read-gap-length 1, segment-mismatches 1, library-type fr-first strand, coverage-search, prefilter-multihits. The initial, unprocessed read counts ranged from 16.1 to 21.7 million per sample, out of which 11.6–17.1 million survived preprocessing and were aligned. All samples had very high mean Sanger 1.9 encoded sequence quality scores (> 39). Gene-wise read counts were collected in R (version 3.3.2) using GenomicAlignments:summarizeOverlaps (version 1.10.1) and, after normalization using variance stabilizing transformation (vst), they were subjected to sample-level quality control: no batch effects were identified. To identify differentially expressed genes between experimental groups, gene-wise read counts were analyzed in R (version 3.4.1) using DESeq 2 (version 1.16.1), employing Wald statistics and FC shrinkage [98].

### Sample preparation for proteomics

HaCaT cells grown in 6 cm dishes were treated with (7x10^5^ cells/dish) MV3- and HAS3-EVs for 24 h. Along with HAS3-EVs, c-Myc inhibitor was also added to the cells with appropriate control samples. The cells were lysed using RIPA buffer (with protease inhibitor cocktail) (Sigma-Aldrich). 10 µg of total protein amounts were digested using a modified FASP protocol, as previously reported [99] .

### Liquid chromatography high-definition tandem mass spectrometry

The samples were analyzed in a randomized order. 300 ng of digested proteins/replicate (three technical replicates per sample) were used in nano-LC-HD-MS^E^ analysis. The nano-LC-HD-MS^E^ analyses were executed as described [100, 101]. Database searches were carried out against UniProtKB/Swiss-Prot reviewed human database (release 2017.10.03 with 20,239 entries) with the Ion Accounting algorithm and using the following parameters: peptide and fragment tolerance, automatic; maximum protein mass, 750 kDa; minimum fragment ions matches per protein, ≥ 7; minimum fragment ions matches per peptide, ≥ 3; minimum unique peptide matches per protein, ≥ 2; primary digest reagent as trypsin; missed cleavages allowed, 2; fixed modification, carbamidomethylation C; variable modifications, deamidation (N, Q) and oxidation of methionine (M); and false discovery rate < 4%.

### Bioinformatic analyses

For differential expression analysis (defining differentially expressed proteins, DEPs), the list was limited to those quantified with a fold change (FC), FC > 1.5 and *P* ≤ 0.05 by analysis of variance for all comparisons. The list of up/downregulated protein changes with their corresponding unique UniProtKB/Swiss-Prot identifiers served as inputs into Ingenuity^®^ Pathway Analysis (IPA) bioinformatics analyses (Qiagen Bioinformatics, Redwood City, CA, USA) and screened for associated canonical pathways. The identified/quantified proteins with their respective relative abundance protein ratios are reported in supplementary Table 1.

### Cell proliferation

4 × 10^4^ cells/well of HaCaT and 6 × 10^4^ cells/well of WM115 were added to a 12 well plate and treated with EVs. During the EV treatment, cells were grown in 5% EV free serum media. The cells were then harvested and counted for a period of 4 days. During treatment with siRNAs, plasmids and inhibitors, the cells were incubated with EVs for an additional 2 days. At the end of 2 days, they were trypsinized and counted.

### Type I collagen 96 well plate invasion assay

Cells were treated with EVs for 24 h and plated as 5 × 10^4^/well on a pre-coated 96 well plate with type I collagen, as per manufacturer’s instructions (#3457-096-K, R&D Systems, MN 55413, USA**)**. 24 h later, invading cells were stained with Calcein-AM in cell dissociation buffer and the resulting fluorescence was measured at 488 nm in a plate reader.

### Cell cycle array

A high throughput ELISA based antibody array (Full moon biosystems, California, USA) was used to check the expression levels of cell cycle proteins (six replicates each) after treatment with EVs. HaCaT cells grown in 6 cm dishes were lysed using RIPA buffer, treated and the resulting lysates were added to the antibody arrays according to the manufacturer’s protocol. The protein expression levels were quantified by Full moon biosystems services.

### Tissue staining

The specimens including 14 benign nevi, 15 dysplastic nevi, 12 in situ melanomas and 16 invasive melanomas were obtained from the archives of the Kuopio University Hospital under the ethical approval of Kuopio University Hospital and The Finnish National Supervisory Authority for Welfare and Health (VALVIRA). For claspin immunohistochemical staining, 5 µm thick formalin-fixed and paraffin-embedded samples were dewaxed and rehydrated. For antigen retrieval, specimens were incubated in 10 mM citrate buffer, pH 6.0 for 10 min at 120 °C in a pressure cooker and then cooled for 15 min. Endogenous peroxidase was blocked with 1% hydrogen peroxide and unspecific staining with 2% milk powder in 0.1 M phosphate buffer. Thereafter, the sections were incubated with claspin polyclonal antibody (Proteintech Antibodies, 23206-1-AP) in a humidity chamber at + 4 °C overnight. The primary antibody was used at 1:600. Thereafter, the samples were incubated with biotinylated anti-rabbit secondary antibody (1:300, Vector laboratories, Burlingame, CA, USA) at room temperature for 1 h, followed by incubation with the ABC reagent. 3, 3′-diaminobenzidine (DAB) was used as a chromogen to visualize the staining and the nuclei were counterstained with Mayer’s hematoxylin. Finally, the samples were dehydrated and mounted in Depex. The stainings were viewed with an Axio Lab.A1 microscope (Carl Zeiss, Microscopy GmbH, Jena, Germany) and the intensities of the claspin staining’s were estimated as follows: negative (0), weak (1), moderate (2) or strong (3).

### Electronic supplementary material

Below is the link to the electronic supplementary material.

Supplementary material 1 (XLSX 492 kb)

Supplementary material 2 (DOCX 11 kb)

Supplementary material 3. Supplementary Fig. 1: **a** Western blots showing expression of CD44, c-Myc, MAPK p42/44, EGFR, NFkB-p65, E-cad, and slug in HaCaT cells treated with MV3- and HAS3-EVs. Actin was used as the loading control. **b**–**i** Immunostaining of slug in HaCaT cells treated with MV3 and HAS3-EVs. Panels **b**–**d**, **f**–**h** show HaCaT cells immunostained for slug with MV3- and HAS3-EVs treatment. **e**, **i** shows enlarged image of the area indicated by a white box in (**c**, **g**). Effect of various concentrations of short HA oligosaccharides HA6 (**j**) and HA8 (**k**) on the binding of EVs originating from HAS3-induced MV3 cells to HaCaT cells. Data represent mean ± S.E. of four independent experiments. *P value < 0.05, **P value < 0.01, one-way ANOVA (Tukey’s test). Scale bars represent 20 µm in B, C, D, F, G, H and 5 µm in E, I. *E-Cad* E-cadherin. (JPEG 1856 kb)

Supplementary material 4. Supplementary Fig. 2: **a** Nanoparticle tracking analysis of EV levels and **b** size distribution secreted from uninduced (C8161-EVs) and induced (C8161-HAS3 EVs) C8161-GFP-HAS3 cells. **c** Effect of melanoma-derived EVs (C8161 cells) on HaCaT cells proliferation. **d** Effect of doxycycline on EV secretion in MV3 (P) and **c** MV3-GFP cells. **e** HA secretion analysis in MV3 cells treated with or without doxycycline, GFP and GFP-mHAS3 D216A transfection. **f** HA secretion analysis in MV3-GFP cells with or without doxycycline. Data represent mean ± S.E. of three independent experiments. (JPEG 1128 kb)

Supplementary material 5. Supplementary Fig. 3: **a** Detailed view of a differential proteomics network demonstrating the involvement of claspin, CLSPN in the cell cycle process. The fold changes with respective *P* values (by ANOVA) and the number of unique peptides identified in proteomics analysis are given by each node. **b** Western blot presenting truncated GFP-ΔHAS3 bands at ~ 35 kDa in MV3 cells; mock = pcDNA3 vector. **c** Effect of HAS2 + 3 siRNA cocktail on HA secretion in MV3 cells. Knocking down endogenous HAS2 and HAS3 in MV3 cells decreased HA secretion significantly while cells transiently transfected with a truncated version of GFP-HAS3 plasmid (GFP-ΔHAS3) did not affect HA secretion. **d** HA secretion in GFP-HAS3 transfected MV3 cells. **P value < 0.01, one-way ANOVA (Tukey’s test). (JPEG 1125 kb)

## Abbreviations

HAS: Hyaluronan synthase
HA: Hyaluronan
HH: Hedgehog signaling
EVs: Extracellular vesicles
CLSPN: Claspin
EMT: Epithelial-to-mesenchymal transition
IHH: Indian hedgehog
