# Supplementary material for: HAS3-induced extracellular vesicles from melanoma cells stimulate IHH mediated c-Myc upregulation via the hedgehog signaling pathway in target cells
Source: Cell Mol Life Sci. 2019 Dec 9;77(20):4093–115. doi: 10.1007/s00018-019-03399-5 (PMC7532973; doi:10.1007/s00018-019-03399-5)
Supplement: Supplementary file 2 — Supplementary material 2 (DOCX 11 kb) [file 18_2019_3399_MOESM2_ESM.docx]

**Supplementary Table 2 Sequence of q-PCR primers used in this study**

| **Q-PCR Primers** | **Sequence (5’-3’)** |
| --- | --- |
| IHH | Forward: AGG CCG GCT TTG ACT GGG TGT ATT  Reverse: GCG GCC GAG TGC TCG GAC TT |
| DHH | Forward: CCG GCT TCG ACT GGG TCT ACT AC  Reverse: GAC CGC CAG TGA GTT ATC AGC TTT |
| SHH | Forward: CCG GCT TCG ACT GGG TGT ACT A  Reverse: CGC CAC CGA GTT CTC TGC TTT |
| GLI1 | Forward: CCA ACT CCA CAG GCA TAC AGG AT  Reverse: CAC AGA TTC AGG CTC ACG CTT C |
| GLI2 | Forward: AAG TCA CTC AAG GAT TCC TGC TCA  Reverse: GTT TTC CAG GAT GGA GCC ACT T |
| GLI3 | Forward: CGC GAC TGA ACC CCA TTC TAC  Reverse: GTG TTG TTG GAC TGT GTG CCA TT |
| SMO | Forward: GCT ACT TCC TCA TCC GAG GAG TCA  Reverse: GGC GCA GCA TGG TCT CGT T |
| PTCH1 | Forward: CCC CTG TAC GAA GTG GAC ACT CTC  Reverse: AAG GAA GAT CAC CAC TAC CTT GGC T |
| GAS1 | Forward: CGT CAT TGA GGA CAT GCT GGC TAT  Reverse: TTC TCC TTG ACC GAC TCG CAG AT |
| MYC | Forward: GCT CCT GGC AAA AGG TCA GA  Reverse: CCA AGA CGT TGT GTG TTC GC |
