# Supplementary figures and images for: HAS3-induced extracellular vesicles from melanoma cells stimulate IHH mediated c-Myc upregulation via the hedgehog signaling pathway in target cells
Source: Cell Mol Life Sci. 2019 Dec 9;77(20):4093–115. doi: 10.1007/s00018-019-03399-5 (PMC7532973; doi:10.1007/s00018-019-03399-5)

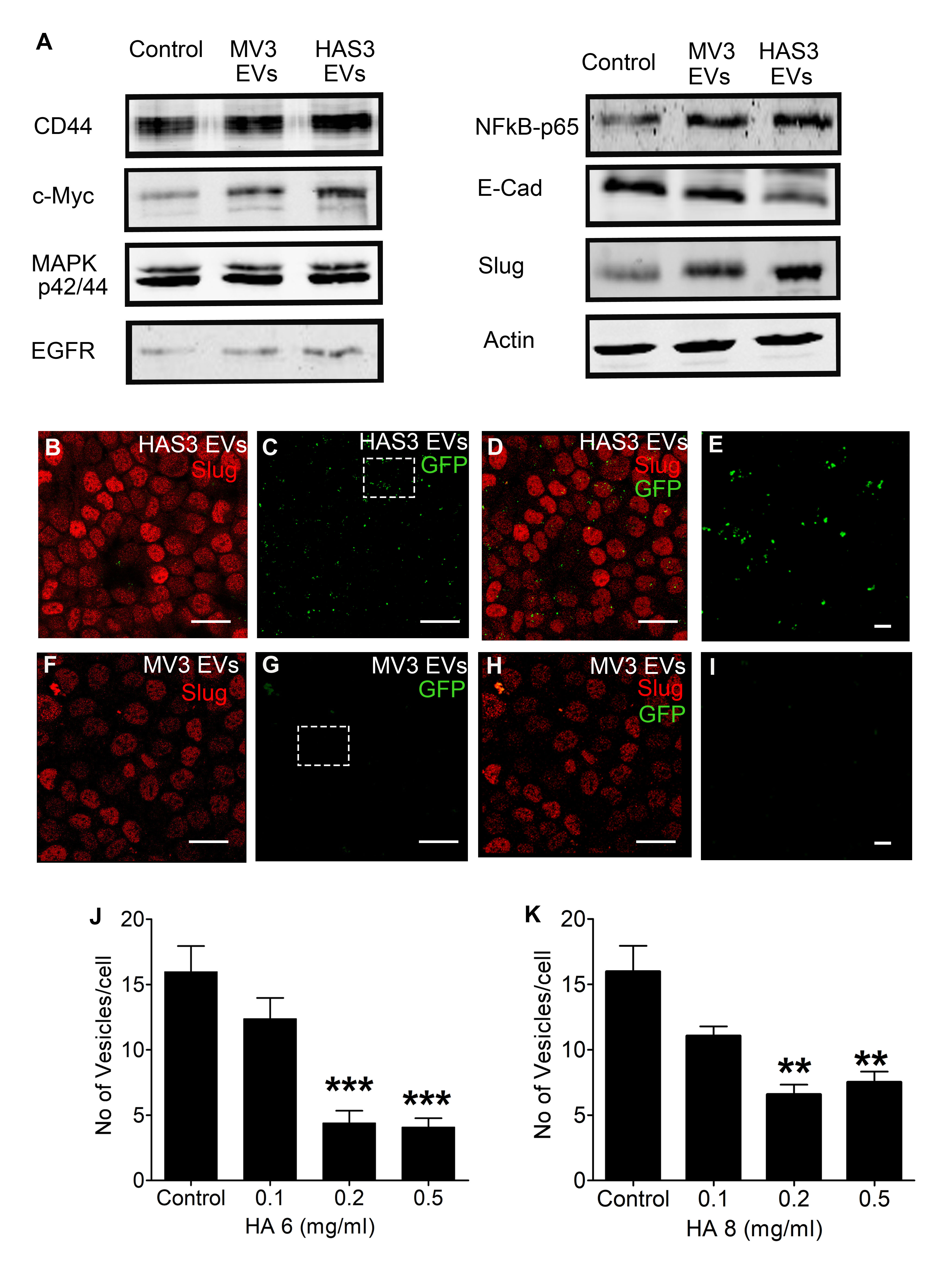

Supplement: Supplementary file 3 — Supplementary material 3. Supplementary Fig. 1: a Western blots showing expression of CD44, c-Myc, MAPK p42/44, EGFR, NFkB-p65, E-cad, and slug in HaCaT cells treated with MV3- and HAS3-EVs. Actin was used as the loading control. b–i Immunostaining of slug in HaCaT cells treated with MV3 and HAS3-EVs. Panels b–d, f–h show HaCaT cells immunostained for slug with MV3- and HAS3-EVs treatment. e, i shows enlarged image of the area indicated by a white box in (c, g). Effect of various concentrations of short HA oligosaccharides HA6 (j) and HA8 (k) on the binding of EVs originating from HAS3-induced MV3 cells to HaCaT cells. Data represent mean ± S.E. of four independent experiments. *P value < 0.05, **P value < 0.01, one-way ANOVA (Tukey’s test). Scale bars represent 20 µm in B, C, D, F, G, H and 5 µm in E, I. E-Cad E-cadherin. (JPEG 1856 kb) [file 18_2019_3399_MOESM3_ESM.jpg]

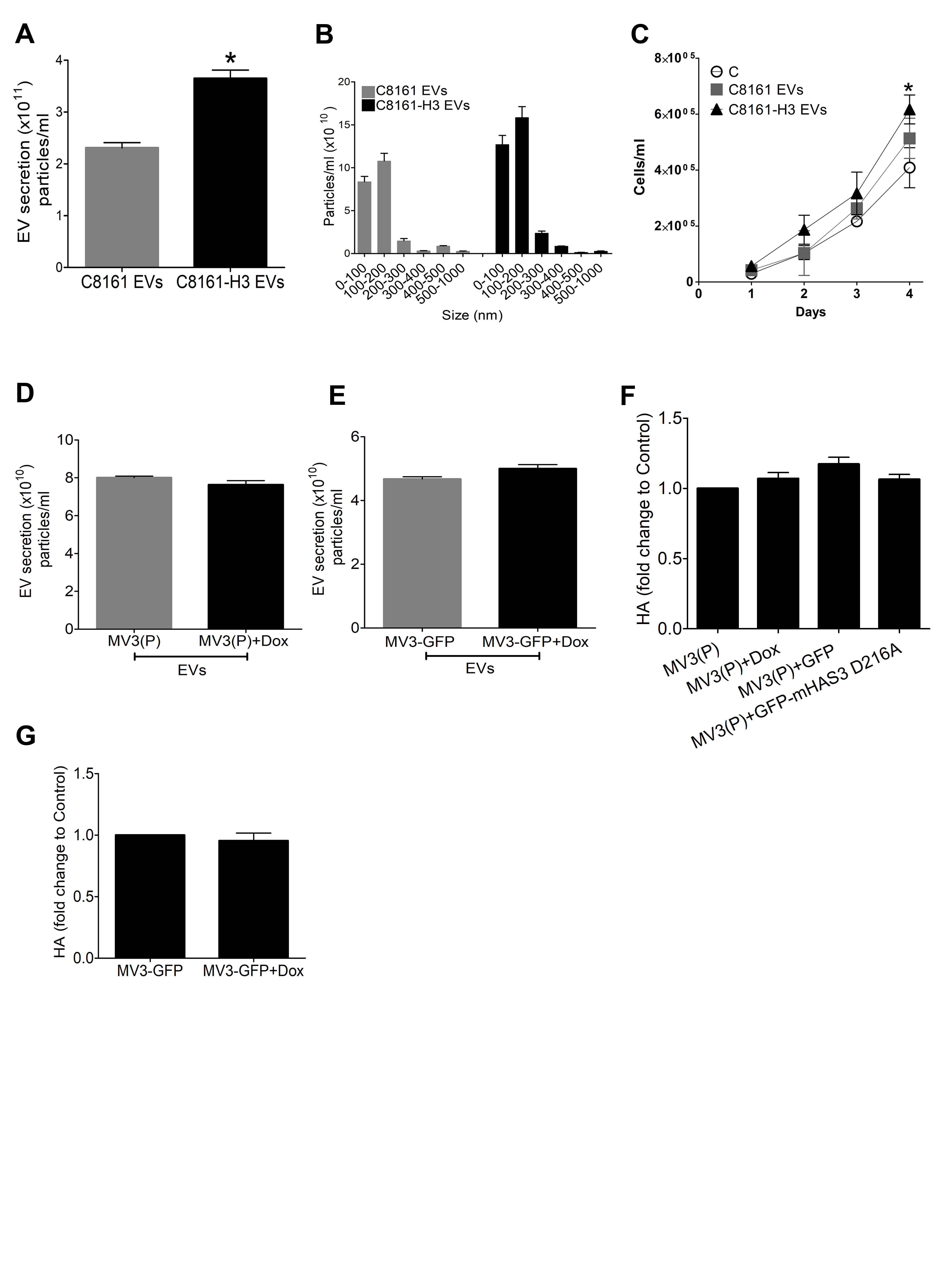

Supplement: Supplementary file 4 — Supplementary material 4. Supplementary Fig. 2: a Nanoparticle tracking analysis of EV levels and b size distribution secreted from uninduced (C8161-EVs) and induced (C8161-HAS3 EVs) C8161-GFP-HAS3 cells. c Effect of melanoma-derived EVs (C8161 cells) on HaCaT cells proliferation. d Effect of doxycycline on EV secretion in MV3 (P) and c MV3-GFP cells. e HA secretion analysis in MV3 cells treated with or without doxycycline, GFP and GFP-mHAS3 D216A transfection. f HA secretion analysis in MV3-GFP cells with or without doxycycline. Data represent mean ± S.E. of three independent experiments. (JPEG 1128 kb) [file 18_2019_3399_MOESM4_ESM.jpg]

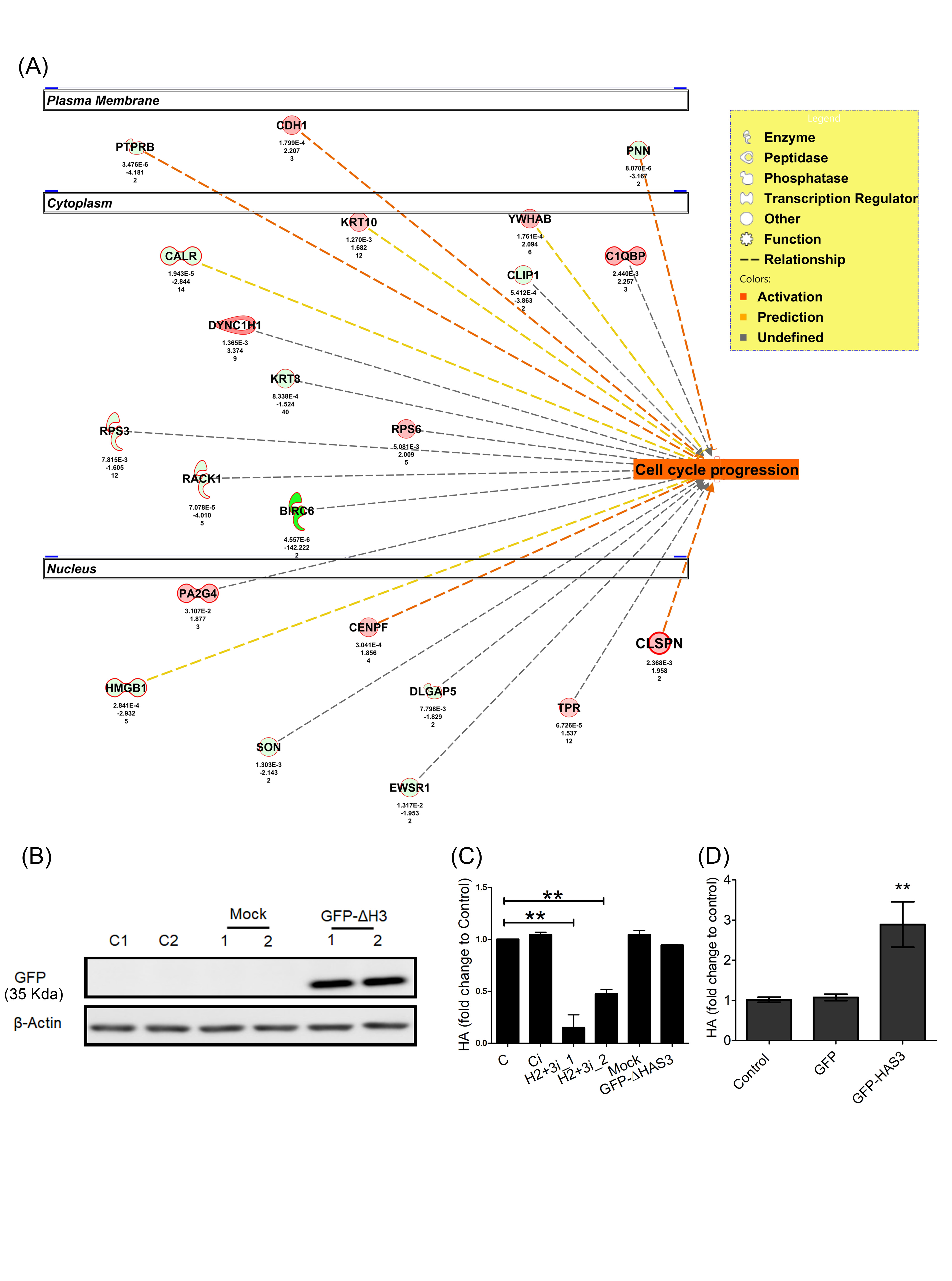

Supplement: Supplementary file 5 — Supplementary material 5. Supplementary Fig. 3: a Detailed view of a differential proteomics network demonstrating the involvement of claspin, CLSPN in the cell cycle process. The fold changes with respective P values (by ANOVA) and the number of unique peptides identified in proteomics analysis are given by each node. b Western blot presenting truncated GFP-ΔHAS3 bands at ~ 35 kDa in MV3 cells; mock = pcDNA3 vector. c Effect of HAS2 + 3 siRNA cocktail on HA secretion in MV3 cells. Knocking down endogenous HAS2 and HAS3 in MV3 cells decreased HA secretion significantly while cells transiently transfected with a truncated version of GFP-HAS3 plasmid (GFP-ΔHAS3) did not affect HA secretion. d HA secretion in GFP-HAS3 transfected MV3 cells. **P value < 0.01, one-way ANOVA (Tukey’s test). (JPEG 1125 kb) [file 18_2019_3399_MOESM5_ESM.jpg]
